# A Systematic Review and Meta-analysis of Psychosocial Interventions to Reduce Drug and Sexual Blood Borne Virus Risk Behaviours Among People Who Inject Drugs

**DOI:** 10.1007/s10461-017-1755-0

**Published:** 2017-04-01

**Authors:** Gail Gilchrist, Davina Swan, Kideshini Widyaratna, Julia Elena Marquez-Arrico, Elizabeth Hughes, Noreen Dadirai Mdege, Marrissa Martyn-St James, Judit Tirado-Munoz

**Affiliations:** 1 National Addiction Centre, Institute of Psychiatry, Psychology and Neuroscience, King’s College London, 4 Windsor Walk, London, SE5 8BB UK; 2 Department of General Practice, University of Melbourne, Parkville, VIC 3010 Australia; 3 Department of Psychiatry and Clinical Psychobiology, School of PsychologyUniversity of Barcelona, 08036 Barcelona, Spain; 4 University of Huddersfield, Huddersfield, HD1 3DH UK; 5 Mental Health and Addiction Research Group, Department of Health Sciences, University of York, York, YO24 2YD UK; 6 School for Health and Related Research, University of Sheffield, Regent Court, 30 Regent Street, Sheffield, S1 4DA UK; 7 Addiction Research Group, IMIM-Institut Hospital del Mar d’Investigacions Mèdiques, Institute of Neuropsychiatry and Addictions, Parc de Salut Mar de Barcelona, 08003 Barcelona, Spain

**Keywords:** People who inject drugs, Psychosocial intervention, Blood borne virus, Injecting risk behaviour, Sexual risk behaviour, Systematic review, Meta-analysis

## Abstract

Opiate substitution treatment and needle exchanges have reduced blood borne virus (BBV) transmission among people who inject drugs (PWID). Psychosocial interventions could further prevent BBV. A systematic review and meta-analysis examined whether psychosocial interventions (e.g. CBT, skills training) compared to control interventions reduced BBV risk behaviours among PWID. 32 and 24 randomized control trials (2000-May 2015 in MEDLINE, PsycINFO, CINAHL, Cochrane Collaboration and Clinical trials, with an update in MEDLINE to December 2016) were included in the review and meta-analysis respectively. Psychosocial interventions appear to reduce: sharing of needles/syringes compared to education/information (SMD −0.52; 95% CI −1.02 to −0.03; I^2^ = 10%; p = 0.04) or HIV testing/counselling (SMD −0.24; 95% CI −0.44 to −0.03; I^2^ = 0%; p = 0.02); sharing of other injecting paraphernalia (SMD −0.24; 95% CI −0.42 to −0.06; I^2^ = 0%; p < 0.01) and unprotected sex (SMD −0.44; 95% CI −0.86 to −0.01; I^2^ = 79%; p = 0.04) compared to interventions of a lesser time/intensity, however, moderate to high heterogeneity was reported. Such interventions could be included with other harm reduction approaches to prevent BBV transmission among PWID.

**Electronic supplementary material:**

The online version of this article (doi:10.1007/s10461-017-1755-0) contains supplementary material, which is available to authorized users.

## Resumen

El tratamiento de sustitución de opiáceos y los programas de intercambio de  jeringuillas han disminuido la transmisión de virus transmitidos por sangre (VTS) entre los usuarios de drogas inyectadas (UDI). Las intervenciones psicosociales podrían prevenir la transmisión de VTS. Mediante una revisión sistemática con meta-análisis, se estudió si las intervenciones psicosociales (p.e. CBT, entrenamiento en habilidades) redujeron los comportamientos de riesgo asociados a los VTS entre UDI, en comparación con las intervenciones control. Se incluyeron 32 y 24 ensayos clínicos aleatorizados en la revisión y meta-análisis, respectivamente (2000-mayo 2015 en MEDLINE, PsycINFO, CINAHL, Colaboración Cochrane y Clinical Trials, con una actualización en MEDLINE hasta diciembre de 2016). Las intervenciones psicosociales disminuyen los siguientes comportamientos de riesgo: compartir agujas/jeringuillas en comparación con la educación/información (DME −0.52; IC del 95%: −1.02, −0.03; I^2^ = 10%; p = 0.04) o en comparación con asesoramiento/pruebas para el VIH (DME −0.24; IC del 95%: −0.44, −0.03; I^2^ = 0%, p = 0.02);  compartir otros utensilios de inyección (DME −0.24; IC del 95%: −0.42, −0.06; I^2^ = 0%; p < 0.01) y el sexo sin protección (DME −0.44; IC del 95%: −0.86, −0.01; I^2^ = 79%; p = 0.04) en comparación con las intervenciones de menor duración/intensidad. Sin embargo, se ha detectado una heterogeneidad de moderada a alta. Tales intervenciones podrían incluirse junto con otros enfoques de reducción de daños para prevenir la transmisión de VTS entre los UDI.

## Introduction

 Among people who inject drugs (PWID), prevalence of the Hepatitis C virus (HCV) and HIV is around 5–90% [1] and <1–50% [2] respectively. While pre-exposure prophylaxis for HIV [3] and treatment for HCV are effective [4], no vaccine prevents HCV. Opiate substitution therapy (OST) and needle exchanges effectively reduce HIV and HCV among PWID [5]. Psychosocial interventions (e.g. motivational interviewing, cognitive behavioural therapy and contingency management) could further decrease blood borne viruses (BBV) by educating PWID about transmission risks and developing strategies to avoid them.

Several systematic reviews and meta-analyses of psychosocial interventions to reduce BBV risk behaviours among drug users have reported modest effects [6–9]. Reviews conclude that harm reduction interventions, especially OST and needle exchange programmes, have reduced injecting risk behaviours, but have not prevented HCV transmission among PWID [5]. In addition, combined substance-use treatment and support for safe injection were most effective in reducing HCV seroconversion among PWID [6]. Interventions that target capability (i.e. individual’s psychological and physical capacity to engage in the activity concerned including having the necessary knowledge and skills), opportunity (i.e. factors outside the individual that make the behaviour possible or prompt it), and motivation are thought to be more effective in addressing behaviour change than interventions that address one or fewer of these components [10]. In partial support of this, two reviews concluded that multi-session psychosocial interventions compared to educational interventions had minimal benefits on injecting risk behaviours [7] and modest benefits on sexual risk behaviours among people who use drugs [8]. In addition, large positive effects were reported compared to minimal interventions for reducing HIV sexual risk behaviours [8]. Despite these promising findings, another review concluded that behavioural interventions (peer-intervention training and counselling interventions) were “unlikely … [*to*] have a considerable effect on HCV transmission” for PWID (p. 176) [9].

PWID are more likely to have BBV than people who use drugs but do not inject [11]. Alongside elevated risks from sharing injecting equipment, some PWID report greater high-risk sexual behaviours, including sex trading, multiple sex partners and sex without a condom, than people who use drugs but do not inject [11–14].

### Objective

This systematic review and meta-analysis sought to examine whether psychosocial interventions could reduce injecting and sexual risk behaviours compared to usual care; education or information; HIV testing and counselling; or interventions of lesser time or intensity (with and without OST).

## Methods

A systematic review with meta-analysis was conducted in accordance with the Preferred Reporting Items for Systematic Reviews and Meta-Analyses [15] and registered with the International Prospective Register of Systematic Reviews (PROSPERO 014:CRD42014012969).

### Search Strategy

The search strategy is described in Table I of the Supplementary Online document. The following databases were searched for relevant randomised controlled trials (RCTs) published from 2000 until 26 May 2015: MEDLINE, PsycINFO, CINAHL and the Cochrane Collaboration, with an update search in MEDLINE to 9 December 2016. Additionally, Clinical Trials databases were searched to identify additional publications. Forward and backward searching of references was conducted and reference lists of recent relevant reviews were cross-checked to verify all relevant RCTs were included in the current systematic review.

### Eligibility

Citations were included if full text was published in English. Studies were eligible if (1) published during 2000–2016; (2) participants were PWID or results presented for PWID; (3) they were RCTs; (4) outcome/s included: (i) any injecting risk behaviour including sharing of needle/syringes or other injecting paraphernalia, and injecting frequency, reported separately or as an aggregated outcome, (ii) any sexual risk behaviour including unprotected sex or number of sexual partners, reported separately or as an aggregated outcome; and (5) psychosocial interventions were compared to a control group, who received usual care, education or information, HIV testing and counselling, “an intervention of lesser time or intensity” [9] (with and without OST).

GG and NM independently assessed all abstracts and potentially eligible full-text manuscripts against eligibility criteria. Where disagreement regarding study inclusion occurred, decisions were reached through referral to additional reviewers, EH, DS, KW.

### Data Extraction

DS, KW, JM, and GG extracted the following data on each study using a checklist: authors, publication year, country, aim of intervention, participants (% PWID, % females, and mean age), intervention delivery setting/staff, intervention description, adherence, description of control interventions, follow-up duration and results (Table 1). These data were verified by a second reviewer and differences resolved through discussion.

**Table 1:** Description of trials included in the systematic review and meta-analyses

Authors	Participants (% female)	Intervention delivery setting/staff	Intervention group description			Control group description		Length of follow-up
			Intervention group/s	Number of sessions	Intervention function/s	Control intervention	Number of sessions	
Abou-Saleh et al. [31]	95 HCV-ve PWID (26%)	Outpatient drug treatment/treatment staff	Enhanced prevention counselling (N = 43)	4 × 40–60 min sessions	Education, enablement	Simple educational counselling (N = 52)	1 × 10 min session	6 months post randomisation
Avants et al. [32]	220 PWID in methadone maintenance treatment (MMT) (69%)	MMT/clinician	Standard care + harm reduction group (N = 108)	12 × 90 min weekly group sessions	Education, enablement, training	Standard care + single HIV risk reduction session (N = 112)	1 × 2 h session	Post intervention
Bertrand et al. [33]	219 PWID that shared drugs or injection equipment (18%)	Not specified/researcher	Motivational intervention (MI) (N = 111)	1 session	Enablement, persuasion	Education Intervention (N = 108)	1 × 30–45 min	6 months post randomisation
Booth et al. [17]	632 PWID (24%)	Residential detoxification/interventionist	Treatment as usual (TAU) + HIV/HCV counselling and education (C&E) (N = 212) TAU + intervention to facilitate treatment entry (N = 209)	2 individual sessions individual session	Education, enablement, training	TAU: HIV/HCV risk assessment screening and referral for testing and counselling (N = 211)	HIV/HCV risk assessment screening and referral for testing and counselling	6 months post randomisation
Dushay et al. [34]	669 drug users [64% injecting] (27%)	Not reported	Ethnic cultural enhanced intervention (n = 453)	5 sessions	Education, enablement, training	AIDS video educational programme (N = 216)	2 video sessions	5–10 months post randomisation (20% interviewed 12 months post randomisation)
El-Bassell et al. [35]	282 HIV-negative drug-using couples (16% PWID) (50%)	Not reported/trained facilitator	Couple-based risk reduction (n = 190) Individual-based HIV risk reduction delivered to male or female drug using partner (n = 183)	7 sessions	Enablement, training	Couple-based wellness promotion (n = 190)	7 sessions	12 months post randomisation
Gagnon et al. [37]	260 PWID (31%)	Needle exchange/computerised intervention	Standard intervention (needle exchanges, psychosocial support and social and health service referrals) + computer-tailored messages (N = 130)	4 sessions	Education, enablement, modelling, persuasion	standard intervention (N = 130)	4 weeks	3 months post intervention
Garfein et al. [38], Purcell et al. [47], Mackesy-Amiti et al. [42, 60, 61]	654 HIV-ve and HCV-ve PWID (34%)	Not reported/trained facilitators	Peer education (N = 431)	6 sessions	Education, enablement, training	Video discussion (N = 423)	6 sessions	6 months post intervention
Gilbert et al. [22]	40 HIV-ve couples who inject drugs (50%)	Needle exchange/facilitators	Couple based HIV risk reduction (20 couples)	3 single-gender group sessions + couple session	Education, modelling, training	Wellness promotion condition (20 couples)	4 group sessions	3 months post intervention
Go et al. [59]	419 index HIV-ve PWID (0%)	Not reported	Peer network-oriented (N = 210)	6 sessions + 3 booster sessions	Education, enablement, training	TAU + HIV pamphlet (N = 209)	n/a	12 months post intervention
Go et al. [30]	455 HIV + ve PWID (0%)	Community intervention education sessions delivered by a trained community mobilizer; Individual HIV knowledge and skill-building group sessions conducted by two facilitators	*Individual level* HIV testing and counselling, plus 2 individual post-test counselling sessions, 2 small group sessions (HIV knowledge and skill-building) + optional dyad session (N = 95) *Community and individual level* Community-wide programme consisting of a 2-part video and a series of 6 HIV education sessions (N = 132)	4 individual sessions + 2 group sessions + optional dyad session 2-part video and a series of 6 HIV education sessions	Education, enablement, training education	*Individual level* HIV testing and counselling (N = 89) *Community level* Standard messages on HIV through village weekly public loudspeakers and educational pamphlets already being provided by community health stations (N = 139)	2 sessions	24 months post randomisation
Hoffman [23]	432 PWID (33%)	Research centre/facilitators	Psychological-communicative behavioral training Peer educator (N = 99) Network members (N = 127)	7 group + 1 individual session + 4 booster meetings	Education, modelling, persuasion, training	Group sessions devoted to areas of interest (N = 92) Network members (N = 114)	8 sessions	24 months post randomisation
Latka et al. [41], Kapadia et al. [58]	418 HCV + ve PWID (24%)	3 research sites/facilitators	Peer-mentoring behavioural intervention (N = 222)	6 sessions	Education, training	Video-discussion (N = 196)	6 sessions	6 months post intervention
Latkin et al. [21]	250 [47% PWID] (39%)	Clinic/indigenous para-professional facilitators	Small-group which encouraged peer outreach (N = 81)	10 group sessions	Enablement, education, training	Equal-attention control condition (N = 36)	10 sessions	6 months post intervention
Latkin et al. [18, 57]	414 networks with 1123 HIV-ve participants [91% PWID (3% in Thailand; 20% in US)	Not reported/facilitator	HIV counselling and testing (C&T) + group peer-education (N = 204 networks)	2 individual + 6 group + 2 booster sessions	Education, enablement, persuasion, training	HIV C&T (N = 210 networks)	2 sessions	Up to 30 months (24 in Thailand) post randomisation
Margolin et al. [43]	90 PWID HIV + ve entering MMT (30%)	MMT/counsellor	Enhanced-MMT (E-MMP) (6 months of daily methadone and weekly individual substance abuse counseling and case management) + HIV Harm Reduction Program (N = 45)	6-sessions	Enablement, persuasion, training	Enhanced-MMT (E-MMP) + active control that included harm reduction components (N = 45)	6 sessions	9 months post randomisation
McMahon et al. [44, 45]	330 HIV-ve drug users [48% PWID] (100%) and male partners	Field office/interventionist	(1) Couple-Based HIV C&T (N = 110) [43% PWID] (2) Women only relationship-focused HIV C&T (N = 104) [51% PWID]	2 sessions 2 sessions	Education, enablement	HIV C&T (N = 116) [51% PWID]	2 sessions	9 months post intervention
Otiashvili et al. [46]	40 drug users [98% PWID] (0%) and drug-free female partners	Research unit/counsellor	MI for drug user and couple, contingency management + naltrexone (n = 20). Female partners invited to attend couples counselling	22 sessions	Enablement, incentivisation	Education sessions. Referrals drug treatment (n = 20)	22 sessions	6 months post intervention
Purcell et al. [47, 56]	966 HIV +ve PWID (36%)	Not reported/peers	Peer mentoring intervention (N = 486)	10 sessions (7 group; 2 individual; 1 ‘peer activity’)	Education, enablement, training	Video discussion (N = 480)	8 sessions	12 months post intervention
Robles et al. [48]	557 PWID (4%)	Assessment facility or drug treatment/nurse	HIV/AIDS risk intervention + counselling + case management (N = 285)	2 sessions 6 sessions	Education, Enablement, training	HIV/AIDS risk intervention (N = 272)	2 sessions	6 months post randomisation
Rotheram-Borus et al. [49] and Hershberger et al. [40]	1116 drug users [65% PWID] (33%)	Field office/counsellors/outreach workers	HIV C&T + HIV prevention programme (group skills focused workshops, individual counselling, outreach/social events) (N = 558; 359 PWID)	2 HIV C&T sessions + 5 sessions (2 group, 1 individual +≥2 structured outreach)	Enablement, training	HIV C&T (N = 559; 364 PWID)	2 sessions	9 months post randomisation
Samet et al. [50]	700 HIV + ve with risky sex and heavy alcohol consumption [60% PWID] (41%)	Hospital setting/peers	Healthy relationships intervention (n = 350; 212 PWID)	2 individual + 3 small group sessions	Education, enablement, modelling, training	Attention control (n = 350; 211 PWID)	2 individual + 3 group sessions	12 months post randomisation
Schroeder et al. [20] and Epstein et al. [36]	81 drug users [96% PWID] (52%)	Research clinic/counsellor	MMT + individual counselling followed by 12 weeks of intervention condition, followed by a 12-week standard treatment (1) weekly CBT + contingent vouchers (CM) (N = 16) (2) weekly CBT + noncontingent vouchers (N = 19) (3) CM plus weekly group therapy (N = 22)	29 weeks (methadone + 5 weekly individual counselling, 12 weeks of intervention, followed by 12 weeks standard treatment	Education, Enablement, Incentivisation Modelling, training	Standard dose of methadone (70–80 mg/day) + + weekly individual counselling (5 weeks) followed by 12 weeks of control condition, followed by a 12-week standard treatment Group therapy + non-contingent vouchers (N = 24)	29 weeks (methadone + 5 weekly individual counselling, 12 weeks of intervention, followed by 12 weeks standard treatment	Post intervention
Stein et al. [51]	109 PWID who drink hazardously (38%)	Research site/social worker	Referrals for substance use and medical treatment + brief MI (N = 60)	2 sessions	Enablement	Referrals for substance abuse and medical treatment provided (N = 49)	n/a	6 months post randomisation
Stein et al. [52]	109 PWID (36%)	Outpatient academic research office/Clinical Psychologist	CBT + pharmacotherapy for depression (N = 53)	8 CBT sessions + 3 pharmacotherapy visits	Education, Enablement, Training	Assessment (N = 56)	Assessment visit	9 months post randomisation
Stein et al. [53]	277 HCV-ve drug users [28% PWID] (37%)	Not stated/interventionist	MI (N = 140)	4 sessions	Education, enablement, persuasion	Leaflet about local resources (N = 137)	n/a	24 months post randomisation
Sterk et al. [29]	68 HIV-ve PWID (100%)	Health intervention project house/interventionists	Enhanced MI (EMI) (N = 20) Enhanced negotiation intervention (ENI) (N = 21)	4 sessions 4 sessions	Education, enablement, training	NIDA standard intervention (N = 27)	2 sessions	6-months (not clear if post intervention or post randomisation)
Strathdee et al. [26] and Vera et al. [55]	584 Sex workers who inject drugs (100%)	Not reported/counsellors	Interactive injection risk and didactic sexual risk intervention (N = 146) Interactive sexual risk and didactic injection risk intervention (N = 148) Interactive Injection and Sexual Risk Intervention) (N = 146)	1 session 1 session 1 session	Education, enablement, modelling, persuasion, training	Didactic injection and sexual risk intervention (N = 144)	1 × 60 min session	12 months post randomisation
Tobin et al. [25]	227 PWID (45%)	Research clinic/not reported	Peer educator intervention (N = 114)	7 sessions (5 group + 1 individual + 1 with participant and enrolled Risk Network Members)	Education, enablement, training	Group information (N = 113)	5 sessions	18 months post intervention
Tucker et al. [54]	145 PWID (26%)	Out-patient clinical and research organization/clinical researcher	Tailored brief behavioural intervention (N = 73)	1 session	Education, enablement	HCV educational leaflet (N = 72)	n/a	1 month post randomisation
Wechsberg et al. [24]	100 PWID (100%)	Inpatient detoxification/psychologist	Woman-focused Intervention (N = 51)	2 sessions	Education, enablement, training	Nutrition intervention (N = 49)	2 sessions	3 months post randomisation
Zule et al. [19]	851 PWID (27%)	Not reported/lay community members	HCV risk reduction MI (n = 423)	6 sessions	Education, enablement, modelling, persuasion	Video HCV educational intervention (n = 428)	6 sessions	12 months post randomisation

### Methodological Quality

Two authors (from GG, DS, JM, JT) independently assessed the trial’s methodological quality across six domains using the Cochrane Risk of Bias tool [16]: sequence generation; allocation concealment; blinding of participants, personnel, and outcome assessors; incomplete outcome data; selective outcome reporting; and other sources of bias (Fig. 2). Assessments were compared to quality assessments from published reviews [5–7, 9], and differences resolved through discussion with a third assessor, EH.

### Intervention Descriptions

Intervention functions were categorised using The Behaviour Change Wheel [10], including: education (increasing knowledge/understanding), e.g. “The 30 min pre-test counseling session provided basic information about…how to reduce the risk of HIV” [17], persuasion (using communication to induce positive/negative feelings/stimulate action) e.g. “facilitators…praise[d] their effective communication strategies” [18] and “reaffirmed commitment to change” [19], incentivisation (creating expectation of reward), e.g. “Contingent vouchers were given when a participant provided a cocaine-negative urine specimen” [20], training (imparting skills), e.g. “skills building to teach personal risk reduction and negotiation” [21] and “technical condom use and syringe disinfection skills” [22], modelling (providing example/s for people to aspire to/imitate), e.g. “model injection and sexual risk reduction behaviors with their risk network members” [23] and “demonstration and rehearsal of syringe cleaning” [19], and enablement (increasing means/reducing barriers) e.g. “women created a personalized risk-reduction plan” [24], “goal-setting for HIV risk reduction” [25] and role play “to help identify barriers to safer injection” [26] (Table 1). Functions that related directly to the intervention’s target behaviour/s were coded. GG and DS independently determined intervention functions. Disagreements were resolved through discussion. Intervention functions assigned to five trials were validated by a behaviour change expert.

### Statistical Analysis

The principal summary measure was the standardized mean difference (SMD). As outcome data were presented as dichotomous or continuous data across included RCTs, odds ratios (OR) were recalculated as SMD to allow data pooling. The standard errors of log OR were converted to standard errors of a SMD by multiplying by the same constant (√3/π = 0.5513). This allowed the standard error for the log OR and hence a confidence interval (CI) to be calculated. For each RCT, the SMD and corresponding 95% CIs for the assessed outcome were retrieved or calculated [27]. Data entry and statistical analysis were performed using Review Manager Software. Where RCTs reported data from various follow-up periods, data from the latest follow-up period were included in the meta-analysis, combining outcomes assessed at multiple time periods. To determine whether RCTs included in the meta-analysis were consistent, the degree of heterogeneity was calculated. *I* ^2^ of 25% was considered low, 50% moderate and 75% high heterogeneity [28]. In the inverse variance method, individual effect sizes were weighted according to the reciprocal of their variance calculated as the square of the standard error.

### Main and Subgroup Analysis

In line with a recent Cochrane review [7] and in an attempt to address the complexity of clinical heterogeneity of interventions, sub-group analyses were conducted to compare psychosocial interventions with (1) treatment as usual; (2) education or information; (3) HIV testing and counselling; and (4) control interventions of lesser time or intensity with and (5) without OST. As follow-up duration may affect intervention effectiveness, further sub-group analyses were conducted where possible, comparing length of time in months from the end of the intervention to the final follow-up of included trials (i.e. ≤3, 4–6, and ≥9 months follow-up).

Where RCTs included in the meta-analyses had more than one intervention group [17, 20, 29], data from the psychosocial intervention most relevant to the aims of this systematic review were compared to the control intervention. For Booth et al. [17], the most relevant intervention condition was considered treatment as usual (TAU) + HIV/HCV counselling and education (C&E), rather than TAU + a therapeutic alliance to facilitate treatment entry. For Sterk et al. [29], the enhanced negotiation intervention (ENI) was considered more relevant than the enhanced motivation intervention (EMI). For Schroder et al. [20], TAU+ weekly CBT + contingent vouchers (CM) (CBT + CM) was considered superior to both weekly CBT + noncontingent vouchers and CM + weekly group therapy, and therefore selected as the intervention condition for the meta-analysis. Go et al. [30] conducted a multi-level intervention using a 2 × 2 (four-arm) factorial design consisting of: (1) standard of care (i.e. HIV testing and counselling); (2) structural-level community stigma reduction programme; (3) individual-level post-test counselling and skill-building support groups; (4) both individual and structural level activities. For the purpose of this systematic review, the individual-level post-test counselling and skill-building support groups will be compared to individual standard of care.

 Random effect models were applied to compare the following outcomes of interest for meta-analysis by type of control intervention, and by type of control intervention and length of follow-up post intervention: any injecting risk behaviour (Fig. 3a; Table 2) including sharing of needle/syringes (Fig. 3b; Table 2) or other injecting paraphernalia (Fig. 3c; Table 2), and frequency of injecting (Fig. 3d; Table 2), reported separately or as an aggregated outcome; and any sexual risk behaviour (Fig. 4a; Table 3) including unprotected sex (Fig. 4b; Table 3) or number of sexual partners (Fig. 4c; Table 3), reported separately or as an aggregated outcome.

**Table 2:** Injecting risk behaviours by control intervention and length of follow-up post-intervention

Outcome	N studies	N participants	Std. mean difference (IV to random to 95 CI)	*p* value for effect	I^2^ (%)
Any injecting risk behaviours by control intervention and length of follow-up post-intervention					
*Subgroups*					
Usual care (4–6 months)	3	641	−0.09 (−0.32 to 0.15)	0.48	26%
Education/information (≤3 months)	1	145	0.00 (−0.35 to 0.36)	0.98	N/A
Education/information (6 months)	2	259	−0.78 (−1.61 to 0.05)	0.07	73%
Education/information (≥9 months)	2	646	−0.41 (−0.74 to −0.08)	0.01	0%
HIV testing and counselling (6 months)	1	557	−0.47 (−0.94 to −0.00)	0.05	N/A
HIV testing and counselling (≥9 months)	2	588	−0.18 (−0.40 to 0.04)	0.11	0%
Intervention lesser frequency or intensity without OST (≥9 months)	2	1389	0.00 (−0.24 to 0.25)	0.98	31%
Intervention lesser frequency or intensity without OST (≤3 months)	2	180	−0.79 (−1.56 to −0.02)	0.04	83%
Intervention lesser frequency or intensity without OST (6 months)	5	1532	−0.32 (−0.55 to −0.10)	0.004	55%
Intervention lesser frequency or intensity with OST (≤3 months)	1	40	0.66 (−0.72 to 2.03)	0.35	N/A
Intervention lesser frequency or intensity with OST (≥9 months)	1	90	0.06 (−0.82 to 0.93)	0.9	N/A
Any injecting risk behaviours by control intervention and length of follow-up post-intervention (overall)	22	6067	−0.29 (−0.42 to −0.15)	<0.0001	61%
Sharing needles/syringes by control intervention and length of follow-up post-intervention					
*Subgroups*					
Usual care (4–6 months)	1	109	−0.53 (−1.12 to 0.07)	0.08	N/A
Education/information (4–6 months)	2	259	−0.57 (−1.30 to 0.16)	0.12	44%
Education/information (≥9 months)	1	419	−0.24 (−1.23 to 0.76)	0.64	N/A
HIV testing and counselling (4–6 months)	1	557	−0.47 (−0.94 to −0.00)	0.05	N/A
HIV testing and counselling (≥9 months)	2	588	−0.18 (−0.40 to 0.04)	0.11	0%
Intervention lesser frequency or intensity without OST (≥9 months)	1	423	0.11 (−0.15 to 0.37)	0.4	N/A
Intervention lesser frequency or intensity without OST (≤3 months)	1	80	−1.19 (−1.67 to −0.72)	<0.00001	N/A
Intervention lesser frequency or intensity without OST (4–6 months)	2	165	−0.65 (−1.12 to −0.17)	0.01	40%
Intervention lesser frequency or intensity with OST (≤3 months)	1	40	0.66 (−0.72 to 2.03)	0.35	N/A
Intervention lesser frequency or intensity with OST (≥9 months)	1	90	−0.60 (−1.17 to −0.03)	0.04	N/A
Sharing needles/syringes by control intervention and length of follow-up post-intervention (overall)	13	2730	−0.43 (−0.69 to −0.18)	0.0003	68%
Sharing other injecting paraphernalia by control intervention and length of follow-up post-intervention					
*Subgroups*					
Education/information (4–6 months)	1	219	−0.42 (−0.98 to 0.14)	0.15	N/A
HIV testing and counselling (4–6 months)	1	557	−0.11 (−0.43 to 0.21)	0.51	N/A
HIV testing and counselling (≥9 months)	2	588	−0.20 (−0.40 to 0.01)	0.06	0%
Intervention lesser frequency or intensity (≤3 months)	1	100	−0.41 (−0.83 to 0.01)	0.05	N/A
Intervention lesser frequency or intensity (4–6 months)	2	902	−0.20 (−0.40 to −0.01)	0.04	0%
Sharing other injecting paraphernalia by control intervention and length of follow-up post-intervention (overall)	7	2366	−0.21 (−0.34 to −0.09)	0.0005	0%
Frequency of injecting by control intervention and length of follow-up post-intervention					
*Subgroups*					
Usual care (4–6 months)	1	423	0.00 (−0.20 to 0.21)	0.96	N/A
Education/information (4–6 months)	1	40	−1.05 (−2.07 to −0.03)	0.04	N/A
HIV testing and counselling (≤3 months)	3	1694	−0.16 (−0.40 to 0.08)	0.2	76%
HIV testing and counselling (4–6 months)	3	1694	−0.16 (−0.40 to 0.08)	0.2	76%
HIV testing and counselling (≥9 months)	3	1694	−0.16 (−0.40 to 0.08)	0.2	76%
Intervention lesser frequency or intensity without OST (≤3 months)	1	100	−0.12 (−0.54 to 0.29)	0.56	N/A
Intervention lesser frequency or intensity without OST (4–6 months)	1	48	−0.75 (−1.35 to −0.16)	0.01	N/A
Intervention lesser frequency or intensity with OST (≤3 months)	1	40	0.09 (−0.61 to 0.79)	0.8	N/A
Frequency of injecting by control intervention and length of follow-up post-intervention (overall)	8	5733	−0.16 (−0.26 to −0.05)	0	63%

**Table 3:** Sexual risk behaviours by control intervention and length of follow-up post-intervention

Outcome	N studies	N participants	Std. mean difference (IV to random to 95 CI)	p value for effect	I^2^ (%)
Any sexual risk behaviours by control intervention and length of follow-up post-intervention					
*Subgroups*					
Education/information (≤3 months)	1	145	−0.21 (−0.56 to 0.15)	0.25	N/A
Education/information (≥9 months)	2	1078	−0.10 (−0.41 to 0.20)	0.51	60%
HIV testing and counselling (≥9 months)	1	174	0.14 (−0.81 to 1.09)	0.77	N/A
Intervention lesser frequency or intensity without OST (≤3 months)	2	180	−0.65 (−0.96 to −0.34)	<0.0001	0%
Intervention lesser frequency or intensity without OST (4–6 months)	1	95	0.19 (−0.26 to 0.64)	0.41	N/A
Intervention lesser frequency or intensity without (OST ≥9 months)	1	966	0.01 (−0.24 to 0.25)	0.96	N/A
Intervention lesser frequency or intensity with OST (≤3 months)	1	40	0.51 (−0.54 to 1.57)	0.34	N/A
Intervention lesser frequency or intensity with OST (≥9 months)	1	90	−0.76 (−1.55 to 0.04)	0.06	N/A
Any sexual risk behaviours by control intervention and length of follow-up post-intervention (overall)	10	2768	−0.19 (−0.39 to 0.01)	0.07	58%
Unprotected sex by control intervention and length of follow-up post-intervention					
*Subgroups*					
Education/information (≥9 months)	1	851	0.03 (−0.18 to 0.24)	0.79	N/A
HIV testing and counselling (≥9 months)	1	174	0.14 (−0.81 to 1.09)	0.77	N/A
Intervention lesser frequency or intensity without OST (≤3 months)		180	−0.65 (−0.96 to −0.34)	<0.0001	0%
Intervention lesser frequency or intensity without OST (4–6 months)	1	48	−0.61 (−1.19 to −0.02)	0.04	N/A
Intervention lesser frequency or intensity without OST (≥9 months)	1	423	0.01 (−0.19 to 0.21)	0.94	N/A
Intervention lesser frequency or intensity with OST (≤3 months)	1	40	0.51 (−0.54 to 1.57)	0.34	N/A
Intervention lesser frequency or intensity with OST (≥9 months)	1	90	−0.60 (−1.17 to −0.03)	0.04	N/A
Unprotected sex by control intervention and length of follow-up post-intervention (overall)	8	1806	−0.27 (−0.54 to −0.01)	0.04	68%
Number of partners by control intervention and length of follow-up post-intervention					
*Subgroups*					
Education/information (≥9 months)	1	227	0.01 (−0.14 to 0.17)	0.89	N/A
Intervention lesser frequency or intensity without OST (4–6 months)	1	48	3.24 (2.36 to 4.12)	<0.00001	N/A
Number of Partners by control intervention and length of follow-up post-intervention (overall)	2	275	0.11 (−0.05 to 0.26)	0.17	98%

## Results

### Study Selection

**Fig. 1:**
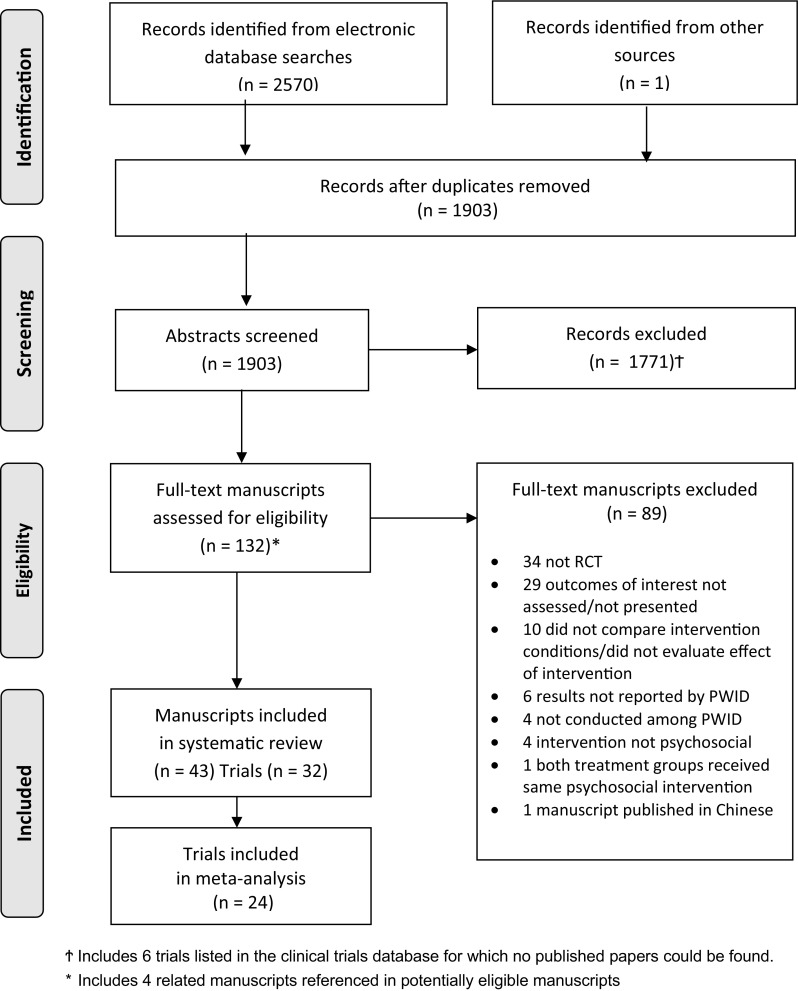
Flowchart. † Includes 6 trials listed in the clinical trials database for which no published papers could be found. *Includes 4 related manuscripts referenced in potentially eligible manuscripts

Database searches to 26 May 2015 resulted in 2493 citations; an additional 77 citations were identified from 1 January 2015 to 9 December 2016 (Fig. 1). One additional manuscript was identified from hand-searching other reviews’ reference lists. After removal of duplicates, 1903 citations remained. In total, 1771 abstracts were excluded as they did not meet eligibility criteria and 132 abstracts were selected for full-text assessment, including four related manuscripts referenced in these selected texts. Eighty-nine articles were excluded as: they were not RCTs (n = 34); outcomes of interest were not assessed/presented (n = 29); outcomes were not presented by PWID (n = 6); number of PWID was not reported (n = 4); or the intervention was not psychosocial (n = 4). Additionally, ten manuscripts were excluded as intervention group outcomes were not compared or did not evaluate the intervention’s effect. One further manuscript was excluded because both treatment arms received the same psychosocial intervention; the intervention arm received a 90-day free OST coupon. One manuscript published in Chinese was excluded [a full list of excluded references available on request].

Forty-two manuscripts from 32 RCTs were eligible [17–26, 29–55]. The meta-analyses included 24 trials [17–22, 24, 25, 29–31, 33, 38, 39, 41, 43, 46–52, 54]. Eight trials were excluded from the meta-analysis for: not providing the number of PWID for control and intervention groups at follow-up [32, 34, 35, 45, 53], only providing risk ratios [37], outcome combined HIV with sexually transmitted infections [26] and data for ‘unsafe injection practices’ was only presented at baseline [23].

### Quality Assessment

**Fig. 2:**
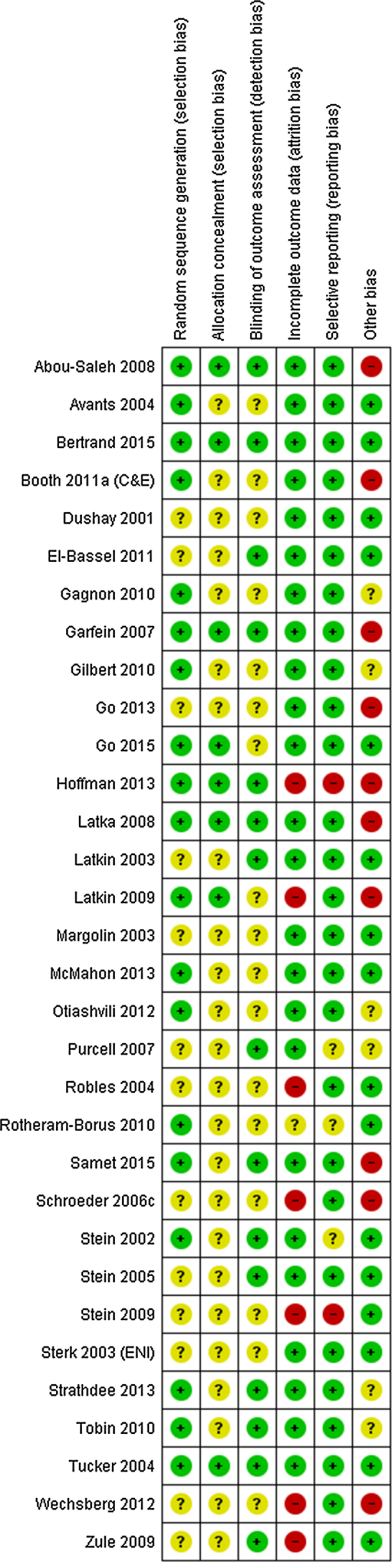
Risk of bias for included trials

Risk of bias varied across RCTs (Fig. 2). Incomplete outcome data was the most common risk of bias, but selective outcome reporting contributed to risk of bias for some trials. Other potential sources included altering randomisation protocols depending on number of participants enrolled on a particular day [41], significant baseline differences between groups in outcome of interest [31], variation in the TAU group across sites [17]; possible cross-over contamination between groups [18, 24, 38, 39, 41, 50], a high proportion of excluded individuals who differed significantly to those included [20], and large variations reported in follow-up period [23].

### Study Characteristics

Characteristics of included RCTs are described in Table 1 (and more detail is presented in the Online Supplementary document Table II). In total, 12,840 participants (35% female; range 0–100%) were enrolled; the majority were PWID (84.5%) (range 16–100%). Most trials (n = 18) were conducted in the US [17, 19–21, 25, 29, 32, 34, 35, 38, 41, 43, 45, 47, 49, 51–53]; three in Russia [23, 24, 50]; two in Canada [33, 37]; two in Vietnam [30, 39]; and one in Kazakhstan [22]; Georgia [46]; Australia [54]; the UK [31]; Mexico [26]; and Puerto Rico [48]. One trial was conducted in both the US and Thailand [18].

Trials included in the systematic review compared psychosocial interventions with usual care (n = 4) [17, 37, 51, 52], education or information (n = 9) [19, 25, 26, 33, 34, 39, 46, 53, 54], HIV testing and counselling (n = 5) [18, 30, 45, 48, 49], interventions of lesser time or intensity with (n = 12) [21–23, 29, 31, 32, 35, 38, 41, 47, 50] and without OST (n = 2) [20, 43].

Of the 32 interventions delivered in the RCTs, most (n = 14) were delivered to individual participants [17, 19, 20, 24, 26, 29, 31, 33, 37, 48, 51–54]; eight were delivered to groups [18, 23, 32, 34, 38, 39, 41, 49] and two to couples [35, 45]. The remaining eight trials delivered interventions in a combination of ways e.g. individual and couples sessions[46]; individual and group sessions [18, 25, 43, 47, 50]; group and couples sessions [22] and one trial provided both individual and structural level activities [30]. For interventions with more than one session, retention or adherence to the intervention ranged from 50% [17] to 95% [29] (further detail provided in the Online Supplementary Table II).

Eight interventions incorporated peer mentoring from an index participant to change the behaviours of other PWID [18, 21, 23, 25, 38, 39, 41, 47, 49]. The majority of interventions contained at least three sessions (n = 25) [18, 20–23, 25, 29–32, 34, 35, 37–39, 41, 43, 46–50, 52, 53], four interventions contained two sessions [17, 24, 44, 51] and three interventions one session [26, 33, 54].

On the whole, interventions were delivered in drug treatment settings including outpatient and hospital clinics [20, 21, 31, 48–50]; methadone maintenance clinics [32, 43]; inpatient or residential detoxification units [17, 24, 54]; needle and syringe exchanges [22, 37] or outreach [49] (settings not mutually exclusive). In addition, the vast majority of studies were delivered by clinic staff in the treatment setting as opposed to researcher delivered (or not specified) (Table 1).

### Outcomes

Various validated and other purposely developed instruments were used to assess injecting behaviour in 32 trials [17–26, 29–35, 37–39, 41, 43, 45–52, 54] and sexual behaviour in 24 trials [17–26, 29–32, 34, 35, 38, 39, 43, 45, 47, 49, 50, 54]. The most common reporting timeframe for outcomes was in the past 30 days(n = 19) [17, 18, 22–26, 29, 32–34, 43, 46, 48–52, 54], followed by the past 3 months (n = 10) [23, 30, 32, 35, 38, 39, 41, 45, 47, 50] or 6 months (n = 3) [21, 25, 53], past week (n = 2) [20, 37] or behaviour at the last sexual encounter or injecting event (n = 1) [19] (answers not mutually exclusive as three trials reported different reporting timeframe for different outcomes) [23, 25, 50].

### Results of Individual RCTs Not Included in the Meta-analyses

Of the eight trials omitted from the meta-analyses, three reported that psychosocial interventions showed greater reductions in injecting risk behaviours than education/information, usual care or HIV testing and counselling [26, 37, 45] and three reported that psychosocial interventions showed greater reductions in sexual risk behaviours than or HIV testing and counselling [32, 35, 45].

### Meta-analyses

#### Any Injecting Risk Behaviour

**Fig. 3:**
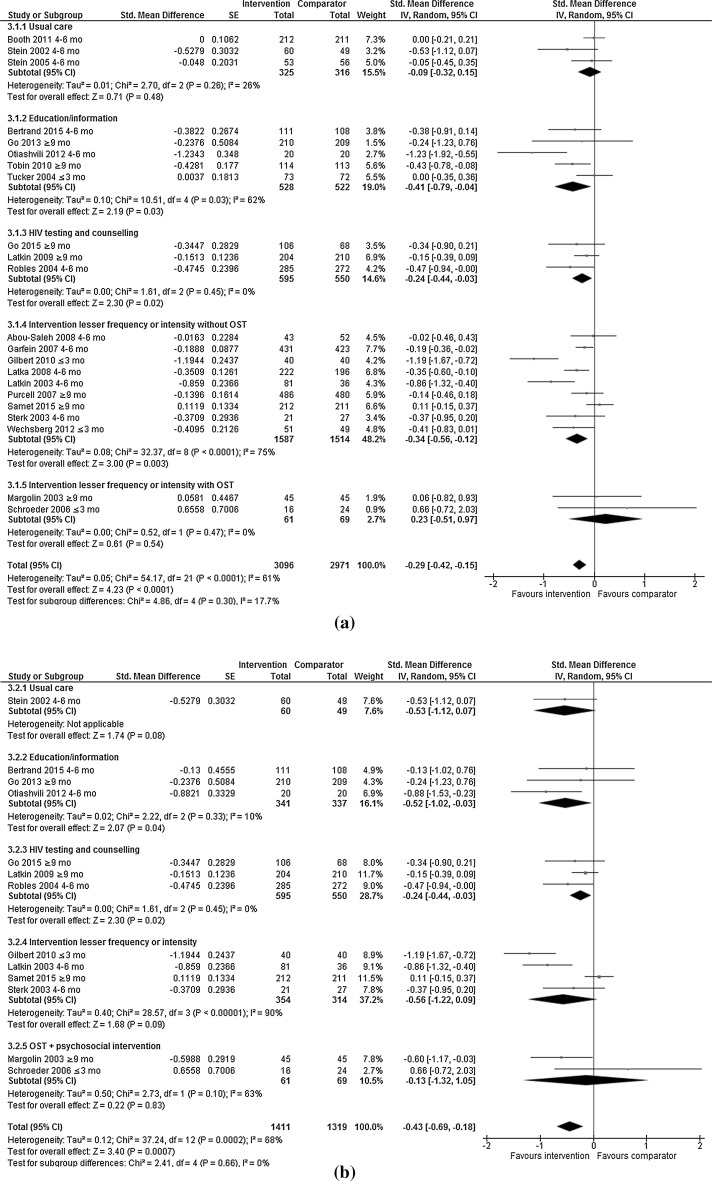

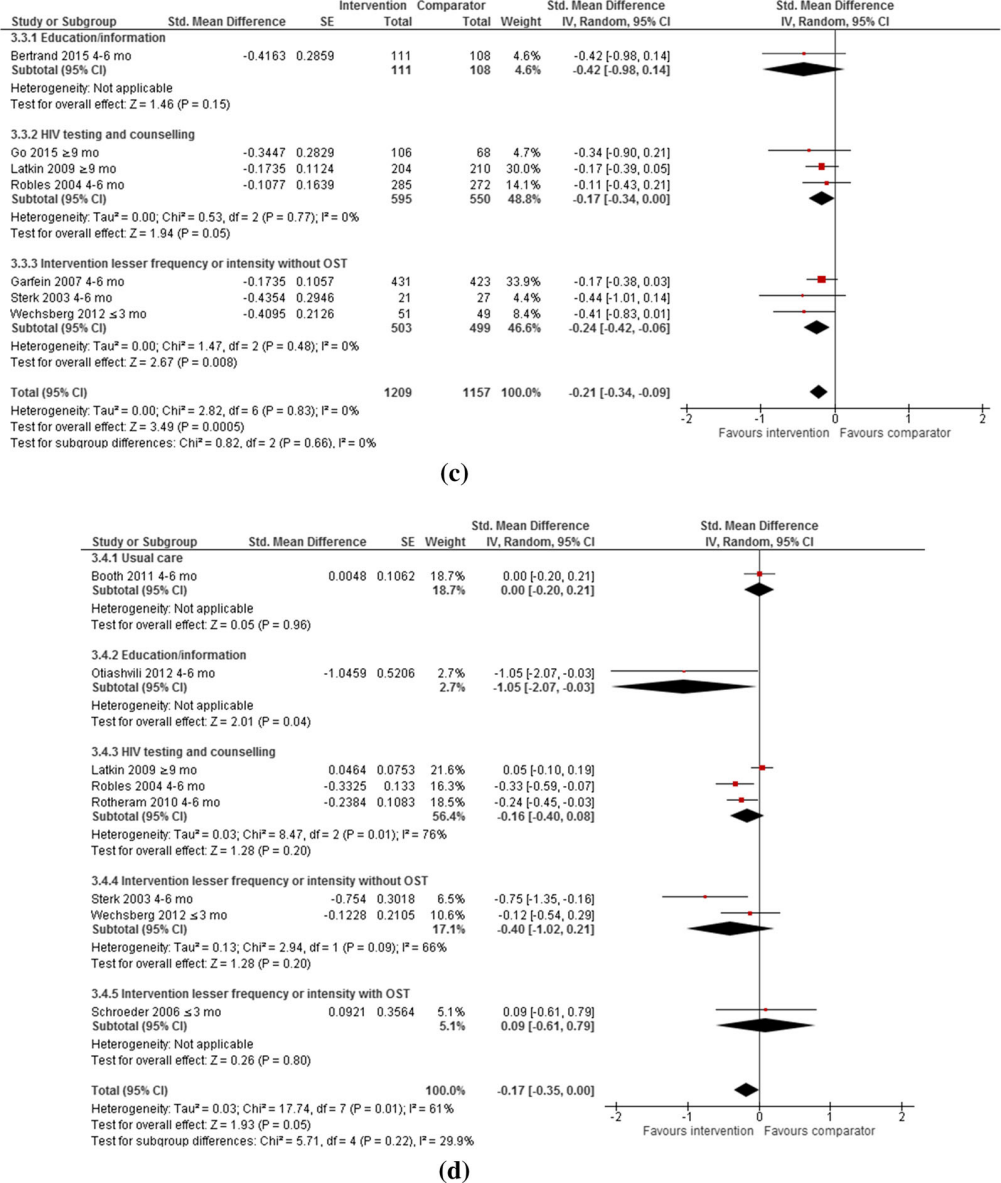
Efficacy of psychosocial interventions versus control interventions in reducing **a** ANY injecting risk behaviours among people who inject drugs, **b** sharing of needles or syringes among people who inject drugs, **c** sharing of other injecting equipment (not needle/syringes) among people who inject drugs, **d** frequency of injecting among people who inject drugs

Twenty-two RCTs assessed any injecting risk behaviour (Fig. 3a). Psychosocial interventions independently reduced injecting risk behaviours more than control interventions in seven trials [21, 22, 25, 38, 41, 46, 48]. A total of 3096 and 2971 PWID were included in the intervention and control groups respectively. Overall, psychosocial interventions showed a greater reduction in any injecting risk behaviour (SMD −0.29; 95% CI −0.42 to −0.15; I^2^ = 61%; p = <0.01) than control interventions (Fig. 3a). Psychosocial interventions also demonstrated greater reductions in any risk behaviours than education/information (SMD −0.41; 95% CI −0.79 to −0.04; I^2^ = 62%; p = 0.03); HIV testing and counselling (SMD −0.24; 95% CI −0.44 to −0.03; I^2^ = 0%; p = 0.02); interventions of a lesser time or intensity (SMD −0.34; 95% CI −0.56 to −0.12; I^2^ = 75%; p < 0.01), but no difference was found when compared with interventions of a lesser time or intensity that included OST (SMD 0.23; 95% CI 0.51–0.97; I^2^ = 0%; p < 0.01) or treatment as usual (SMD −0.09; 95% CI −0.32 to 0.15; I^2^ = 26%; p = 0.54). Where outcomes were assessed ≤3 or 4–6 months post-intervention, psychosocial interventions reduced any injecting risk behaviour when compared with interventions of lesser time or intensity. Where outcomes were compared ≥9 months post-intervention, psychosocial interventions reduced any injecting risk behaviour more than interventions that provided education/information alone (Table 2). Heterogeneity was moderate in psychosocial interventions compared to education/information (I^2^ = 62%), possibly due to the variations in the mode of delivery and intervention components (Table 1). The education/information interventions in the control conditions included a pamphlet compared to a 6 session education/enablement intervention [39] and ranged from comparing a one-session education intervention with a one-session motivational intervention [33], to comparing 22 education sessions with referrals to drug treatment with a 22-week intervention including motivational interviewing counseling sessions for both the male participant and the couple, monetary incentives for drug abstinence, and research-supported detoxification followed by naltrexone treatment [46]. There was high heterogeneity in the analysis of psychosocial interventions compared to interventions of a lesser time or intensity (without OST) (I^2^ = 75%) for similar reasons to those mentioned above. Six trials included equal-attention control conditions ranging from 2 [24] to 10 [41] sessions, and three included control interventions with fewer sessions, ranging from four versus one session [31] to ten versus eight sessions [47] (Table 1). All but one trial [31] had at least two sessions in the control and/or intervention conditions. One trial compared a two-session woman focused intervention with a two-session nutritional intervention [24]. The variation in intervention duration and content across conditions contributes to the high heterogeneity.

#### Sharing Needles and Syringes

Thirteen RCTs assessed sharing of needles/syringes (Fig. 3b). Psychosocial interventions reduced this behaviour more than the control interventions in five of those trials [21, 22, 43, 46, 48]. A total of 1411 and 1315 PWID were included in the intervention and control groups respectively. Overall, psychosocial interventions reduced the sharing of needle/syringes (SMD −0.43; 95% CI −0.69 to −0.18; I^2^ = 68%; p < 0.01) compared with control interventions. Psychosocial interventions reduced needle and syringe sharing compared with education/information (SMD −0.52; 95% CI −1.02 to −0.03; I^2^ = 10%; p = 0.04); or HIV testing and counselling (SMD −0.24; 95% CI −0.44 to −0.03; I^2^ = 0%; p = 0.02); but no difference was found when compared with interventions of a lesser time or intensity (SMD −0.56; 95% CI −0.12 to 0.09; I^2^ = 90%; p = 0.09), interventions of a lesser time or intensity that included OST (SMD −0.13; 95% CI −1.32,1.05; I^2^ = 63%; p = 0.83) or treatment as usual (SMD −0.53; 95% CI −1.12 to 0.07; p = 0.08; one trial). Where outcomes were assessed ≤3 or 4–6 months post-intervention, psychosocial interventions reduced needle and syringe sharing more than interventions of lesser time or intensity. Where outcomes were assessed 4–6 months post-intervention, a greater reduction in any injecting risk behaviour was found for psychosocial interventions compared with HIV testing and counselling (Table 2). There was moderate and high heterogeneity in the analysis of psychosocial interventions compared to interventions of a lesser time or intensity with (I^2^ = 63%) and without OST (I^2^ = 90%), again potentially explained by the differences in intervention content and delivery. The two trials that compared psychosocial interventions with OST to interventions of a lesser time/intensity with OST varied in length of OST treatment. Both included a 12-week psychosocial intervention, however, the trial where methadone was prescribed for six months independently reduced needle and syringe sharing [43] and the trial that prescribed methadone for three months did not [20]. Four trials compared psychosocial interventions with interventions of a lesser time or intensity without OST, two delivered to couples [22] or encouraged peer outreach [21] independently reduced needle and syringe sharing, whereas those delivered to individuals on their own or in groups did not [29, 50].

#### Sharing Other Injecting Paraphernalia

Seven RCTs assessed sharing of injecting paraphernalia (other than needles/syringes) (Fig. 3c). None independently found the psychosocial intervention to be more efficacious than the control interventions. A total of 1209 and 1157 PWID were included in the intervention and control groups respectively. Overall, psychosocial interventions showed greater reductions in the sharing of other injecting paraphernalia (SMD −0.21; 95% CI −0.34 to −0.09; I^2^ = 0%; p < 0.01) compared with control interventions. Psychosocial interventions reduced the sharing of other injecting paraphernalia more than interventions of a lesser time or intensity without OST (SMD −0.24; 95% CI −0.42 to −0.06; I^2^ = 0%; p < 0.01); but no differences were found when compared with education/information (SMD −0.42; 95% CI −0.98 to 0.14; p = 0.15; one trial); and HIV testing and counselling (SMD −0.17; 95% CI −0.34 to 0.00; I^2^ = 0%; p = 0.05). Where outcomes were compared 4–6 months post-intervention, psychosocial interventions significantly reduced sharing of other injecting paraphernalia compared with interventions of lesser time or intensity (Table 2).

#### Injecting Frequency

Eight RCTs assessed frequency of injecting (Fig. 3d). Psychosocial interventions independently reduced frequency of injecting compared with the control interventions in four trials [29, 46, 48, 49]. A total of 1168 and 1177 PWID were included in the intervention and control groups respectively. Overall, psychosocial interventions showed no difference in reducing frequency of injecting (SMD −0.17; 95% CI −0.35 to 0.00; I^2^ = 61%; p = 0.05). Psychosocial interventions significantly reduced the frequency of injecting compared with education/information (SMD −1.05; 95% CI −2.07 to −0.03; p = 0.04; one trial); but no difference was found when compared with interventions of a lesser time or intensity with (SMD 0.09; 95% CI −0.61 to 0.79; I^2^ = 76%; p = 0.20; one trial) and without OST (SMD −0.46; 95% CI −1.02 to 0.21; I^2^ = 66%; p = 0.80); HIV testing and counselling (SMD −0.16; 95% CI −0.40 to 0.08; I^2^ = 76%; p = 0.20) and treatment as usual (SMD −0.00; 95% CI −0.20 to 0.21; p = 0.96; one trial). Where outcomes were compared 4–6 months post-intervention, the frequency of injecting significantly reduced for participants receiving psychosocial interventions compared with participants receiving education/information (Table 2). There was moderate to high heterogeneity in the analysis comparing psychosocial interventions to HIV testing and counselling (I^2^ = 76%) and to interventions of a lesser time/intensity with (I^2^ = 66%) and without OST (I^2^ = 66%), again potentially explained by the differences in intervention content and delivery described above. All HIV testing and counselling intervention control groups received two sessions compared to the intervention conditions that ranged from seven [49] to ten sessions [18] in comparison.

#### Any Sexual Risk Behaviour

**Fig. 4:**
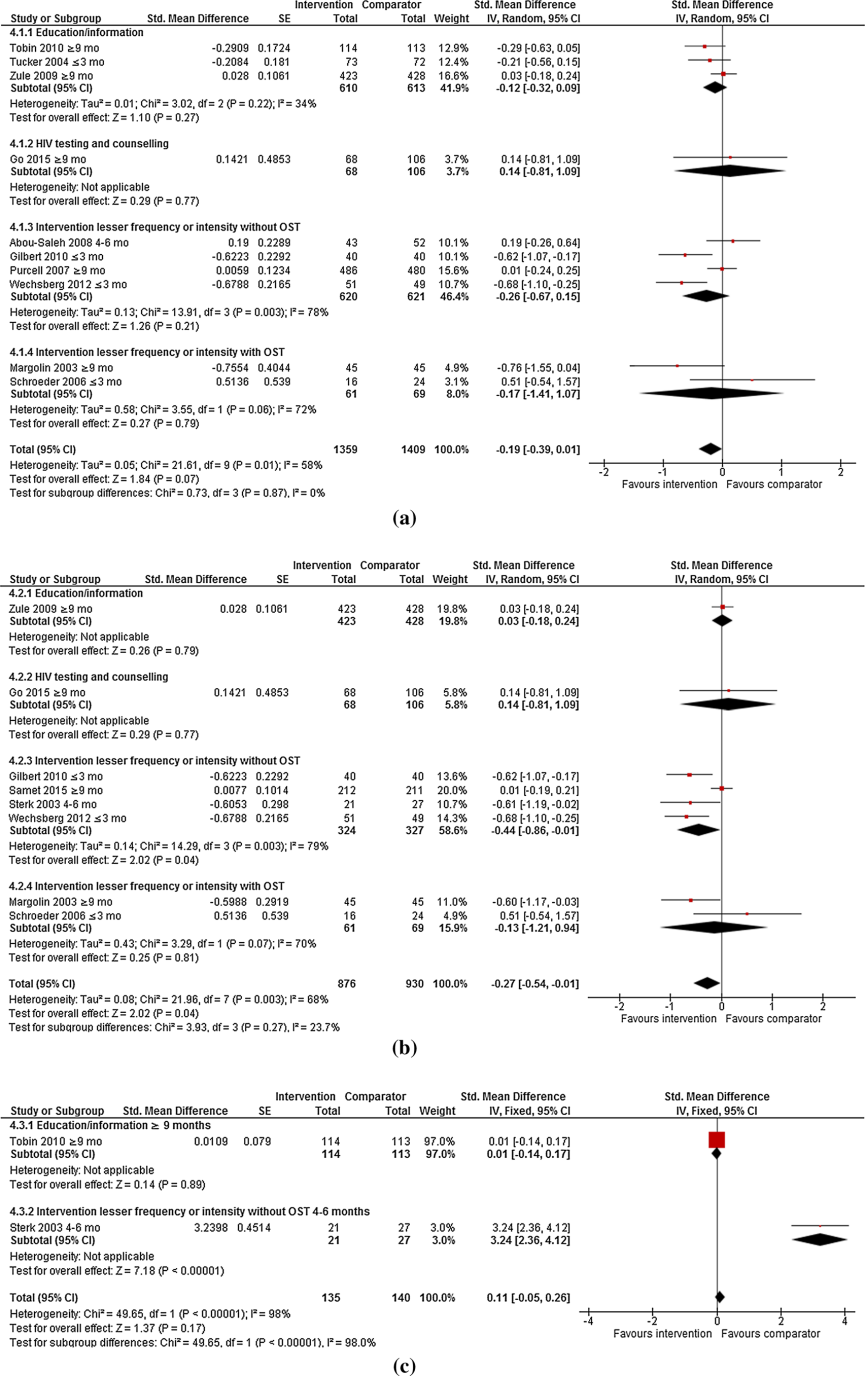
Efficacy of psychosocial interventions versus control interventions in reducing **a** ANY sexual risk behaviours among people who inject drugs, **b** unprotected sex among people who inject drugs, **c** number of sexual partners among people who inject drugs

Ten RCTs assessed any sexual risk behaviour (Fig. 4a). Psychosocial interventions were independently more likely to reduce any sexual risk behaviour than the control interventions in two trials [22, 24]. A total of 1359 and 1409 PWID were included in the intervention and control groups respectively. Overall, psychosocial interventions reduced any sexual risk behaviour compared with control interventions (SMD −0.19; 95% CI −0.39, 0.01; I^2^ = 58%; p = 0.07). Psychosocial interventions showed no difference in reducing any sexual risk behaviours compared with education/information (SMD −0.12; 95% CI −0.32 to 0.09; I^2^ = 34%; p = 0.27), interventions of a lesser time or intensity with (SMD −0.26; 95% CI −0.67 to 0.15; I^2^ = 78%; p = 0.21); and without OST (SMD −0.17; 95% CI −1.41,1.07; I^2^ = 72%; p = 0.79); and HIV testing and counselling (SMD 0.14; 95% CI −0.81,1.09; p = 0.77; one trial). Where outcomes were compared ≤3 months post-intervention, psychosocial interventions reduced any sexual risk behaviour compared with interventions of a lesser time or intensity (without OST) (Table 3). The high heterogeneity in the analysis comparing psychosocial interventions to interventions of a lesser time or intensity with (I^2^ = 78%) and without OST (I^2^ = 72%) has already been discussed.

#### Unprotected Sex

Eight RCTs assessed unprotected sex (Fig. 4b). Psychosocial interventions were independently more effective than the control interventions in four trials [22, 24, 29, 43]. A total of 876 and 930 PWID were included in the intervention and control groups respectively. Overall, psychosocial interventions reduced unprotected sex more than control interventions (SMD −0.27; 95% CI −0.54 to −0.01; I^2^ = 68%; p = 0.04). Psychosocial interventions reduced unprotected sex compared with interventions of a lesser time or intensity (without OST) (SMD −0.44; 95% CI −0.86 to −0.01; I^2^ = 79%; p = 0.04); but no difference was reported when compared with education/information (SMD 0.03; 95% CI −0.18 to 0.24; p = 0.79; one trial), interventions of a lesser time or intensity with OST (SMD −0.13; 95% CI −1.21 to 0.94; I^2^ = 70%; p = 0.81); and HIV testing and counselling (SMD 0.14; 95% CI −0.81,1.09; p = 0.77; one trial). Where outcomes were compared ≤3 and 4–6 months post-intervention, psychosocial interventions reduced unprotected sex compared to interventions of a lesser time or intensity (without OST). Where outcomes were assessed ≥9 months post-intervention, psychosocial interventions reduced unprotected sex compared with interventions of a lesser time or intensity (with OST) (Table 3).

#### Number of Sexual Partners

Two RCTs assessed number of sexual partners (Fig. 4c). A total of 135 and 140 PWID were included in the intervention and control groups respectively. There was no difference between psychosocial interventions and education/information in reducing the number of sexual partners (SMD 0.01; 95% CI −0.14 to 0.17; one trial). Interventions of a lesser time or intensity (without OST) reduced the number of sexual partners compared with psychosocial interventions (SMD 3.24; 95% CI 2.36,4.12; one trial).

## Discussion

The aim of the review and meta-analysis was to identify and evaluate the impact of psychosocial interventions designed to reduce injecting risk and sexual risk behaviours among PWID. A total of 24 trials were included in the meta-analysis. Overall, psychosocial interventions reduced some of the target injecting (sharing of needle and syringes and other injecting paraphernalia) and sexual risk behaviour (unprotected sex) outcomes among PWID when compared with control conditions. More specifically, the meta-analysis found that psychosocial interventions reduced the sharing of needles and syringes compared to education/information or HIV testing and counselling, reduced the sharing of other injecting paraphernalia compared to interventions of a lesser time or intensity, reduced the frequency of injecting compared to one trial of education/information, and reduced unprotected sex compared to interventions of a lesser time or intensity. Although psychosocial interventions targeted injecting risk behaviours rather than a reduction in injecting behaviour per se, one trial reported a significant effect (p = 0.05) with regards to reduced frequency of injecting. Psychosocial interventions were no more likely than control interventions to reduce the number of sexual partners. However, only two trials were pooled in this specific meta-analysis, and many participants reported being in a steady relationship. Interestingly, they also reported a reduction in unprotected sex, a factor which may be more important in reducing BBV transmission than the number of sex partners [25, 29].

Using data on outcomes collected at nine-months or more post-intervention, the meta-analyses found psychosocial interventions produced more reported behaviour change than control interventions, suggesting that maintenance or booster sessions may be required to sustain positive behaviour change.

One study found stronger intervention effects for those who had known their HCV-positive status for at least six months, but not for those who had known their HCV-positive status for more than 12 months [41], suggesting a window of opportunity may exist following HCV diagnosis to address transmission risks.

Overall and regardless of intervention or control content, 16 of the 32 trials included in the systematic review reported greater reductions in injecting or sexual risk behaviours in participants in the intervention group compared to the control group [21, 22, 24–26, 29, 32, 35, 37, 38, 41, 43, 45, 46, 48, 49]. Only two trials in the review (with small sample sizes) included contingency management (incentivisation). One of these trials reported greater reductions in injecting risk behaviours in the intervention group (22 sessions of motivational interviewing for the male participant and couple (female partner drug-free) plus contingency management and naltrexone) compared to the control group (22 sessions of education, including referrals to a detoxification programme and aftercare that may or may not have included naltrexone) [46]. The other reported no significant difference in injecting or sexual risk behaviours between the intervention (29 week intervention including 12 weeks of CBT and contingent vouchers (as well as standard care: methadone + 5 weekly individual counselling, followed by 12 weeks standard treatment) and control groups (29 week standard care intervention (same as intervention group) including 12 weeks of group therapy and non-contingent vouchers) [20] (Table 1). Only three of the seven trials [18, 19, 26, 33, 37, 43, 53] of psychosocial interventions including motivational interviewing found greater reductions in some injecting and sexual risk behaviours [26, 37, 43]. As these three interventions varied in content and participant group (e.g. one session interactive session for female sex workers [26]; computerised intervention (69% male) [37]; and PWID entering OST (70% male) [43]; results about the effectiveness of specific intervention functions (e.g. incentivisation or persuasion) in reducing BBV risk behaviours among PWID are inconclusive.

### Limitations

Limitations include the low number of studies for inclusion in some of the sub-group analyses of behavioural outcomes and intervention delivery modes. In addition, there was heterogeneity in terms of the interventions studied and their duration, as well as differences in sample sizes and characteristics, length of follow-up, and assessment methods used to determine risk behaviours. This lack of consistency across studies may have contributed to the moderate levels of heterogeneity noted in the meta-analyses. The most common risk of bias in included RCTs was selective outcome reporting and possible cross-over contamination between groups. A further limitation is that authors of the eight trials not included in the meta-analysis were not contacted to determine whether they could supply the additional data required to include the trial in the meta-analysis. It is acknowledged that this could have resulted in a potential source of bias in the findings. These limitations need to be considered when interpreting the results.

## Conclusions

Whilst indications from the meta-analysis suggest that psychosocial interventions (when compared to control) reduce risk taking behaviour outcomes, more research is needed. The findings highlight the difficulty and complexity involved in attempting to examine the effectiveness of interventions that include different content and functions, modes of delivery, dosage and number of sessions. This heterogeneity in both the control and intervention conditions resulted in challenges to fully interpret the findings. It will be important to determine what types of psychosocial interventions work for whom and in what settings [8]. Our findings suggest that psychosocial interventions could boost the impact of current harm reduction interventions delivered as routine care and could be included with other harm reduction approaches, including OST and needle and syringe exchange, to reduce BBV transmission risks among PWID. Further trials should address some of the limitations in terms of target populations, dose and frequency and timing of outcome measures.

## Electronic supplementary material

Below is the link to the electronic supplementary material.

Supplementary material 1 (DOCX 55 kb)
