# Supplementary material for: A Systematic Review and Meta-analysis of Psychosocial Interventions to Reduce Drug and Sexual Blood Borne Virus Risk Behaviours Among People Who Inject Drugs
Source: AIDS Behav. 2017 Apr 1;21(7):1791–811. doi: 10.1007/s10461-017-1755-0 (PMC5491643; doi:10.1007/s10461-017-1755-0)
Supplement: Supplementary file 1 — Supplementary material 1 (DOCX 55 kb) [file 10461_2017_1755_MOESM1_ESM.docx]

**Table I. Description of search terms**

| **Database** | **Keywords** |
| --- | --- |
| MEDLINE | Key words for population: people who injection; people who inject drugs; PWID; Substance abuse; intravenous substance abuse; injecting drug use; intravenous drug use; IDU; injection drug use; injector; IVDU; intravenous drug abuse; injecting drug abuse; injection drug abuse.  Key words for BBV: Blood-borne pathogens, BBV , blood borne virus, blood borne bacteria, HIV infection, human immunodeficiency virus, HIV, Hepatitis C, Hepatitis C virus, hep c, HCV, Hepacivirus, Hepatitis B, Hepatitis B virus, HBV.  Key words for intervention: psychosocial *adjacent to* intervention, prevention, support, rehabilitation, treatment, or therapy; psychological *adjacent to* therapy, treatment, or intervention; CBT; cognitive *adjacent to* therapy, behaviour therapy, intervention, or remediation; behaviour *adjacent to* therapy, control, modification, change, or intervention; Psychotherapy; Counseling; Sscial support; motivational *adjacent to* intervention, interview, or support; brief advice; brief intervention; family *adjacent to* therapy, intervention, or counselling, couple *adjacent to* therapy, intervention, or counselling; peer *adjacent to* therapy, intervention, or counselling; talking therapy; contingency management; psychoeducation; route transition; network therapy.  Key words for outcomes: Risk *adjacent to* taking, reduction, protection, minimisation, prevention, decrease, avoidance, or behaviour; harm *adjacent to* reduction, protection, minimisation, prevention, decrease, avoidance; unsafe injecting; needle sharing; intravenous injection; injecting *adjacent to* equipment, paraphernalia, or risk; sexual risk behaviour; unsafe sex; multiple sexual partners; multiple casual partners; one time sex encounter; sex holiday; casual sex; casual partner; non-regular sex partner; unprotected intercourse; unprotected sex; condomless *adjacent to* sex or intercourse; condom free *adjacent to* sex or intercourse; barebacking; bareback sex; bug chase; anal intercourse; anal sex; condom; condom use; safe sex; health *adjacent to* knowledge, attitudes, or practice, or behaviour; transmission knowledge or understanding. |
| PsycINFO | Key words for population: people who injection; people who inject drugs; PWID; Substance abuse; intravenous substance abuse; injecting drug use; intravenous drug use; IDU; injection drug use; injector; IVDU; intravenous drug abuse; injecting drug abuse; injection drug abuse.  Key words for BBV: Blood-borne pathogens, BBV , blood borne virus, blood borne bacteria, HIV infection, human immunodeficiency virus, HIV, Hepatitis C, Hepatitis C virus, hep c, HCV, Hepacivirus, Hepatitis B, Hepatitis B virus, HBV.  Key words for intervention: psychosocial *adjacent to* intervention, prevention, support, rehabilitation, treatment, or therapy; psychological *adjacent to* therapy, treatment, or intervention; CBT; cognitive *adjacent to* therapy, behaviour therapy, intervention, or remediation; behaviour *adjacent to* therapy, control, modification, change, or intervention; Psychotherapy; Counseling; Sscial support; motivational *adjacent to* intervention, interview, or support; brief advice; brief intervention; family *adjacent to* therapy, intervention, or counselling, couple *adjacent to* therapy, intervention, or counselling; peer *adjacent to* therapy, intervention, or counselling; talking therapy; contingency management; psychoeducation; route transition; network therapy.  Key words for outcomes: Risk *adjacent to* taking, reduction, protection, minimisation, prevention, decrease, avoidance, or behaviour; harm *adjacent to* reduction, protection, minimisation, prevention, decrease, avoidance; unsafe injecting; needle sharing; intravenous injection; injecting *adjacent to* equipment, paraphernalia, or risk; sexual risk behaviour; unsafe sex; multiple sexual partners; multiple casual partners; one time sex encounter; sex holiday; casual sex; casual partner; non-regular sex partner; unprotected intercourse; unprotected sex; condomless *adjacent to* sex or intercourse; condom free *adjacent to* sex or intercourse; barebacking; bareback sex; bug chase; anal intercourse; anal sex; condom; condom use; safe sex; health *adjacent to* knowledge, attitudes, or practice, or behaviour; transmission knowledge or understanding. |
| CINAHL | Key words for population: people who injection; people who inject drugs; PWID; Substance abuse; intravenous substance abuse; injecting drug use; intravenous drug use; IDU; injection drug use; injector; IVDU; intravenous drug abuse; injecting drug abuse; injection drug abuse.  Key words for BBV: Blood-borne pathogens, BBV , blood borne virus, blood borne bacteria, HIV infection, human immunodeficiency virus, HIV, Hepatitis C, Hepatitis C virus, hep c, HCV, Hepacivirus, Hepatitis B, Hepatitis B virus, HBV.  Key words for intervention: psychosocial *adjacent to* intervention, prevention, support, rehabilitation, treatment, or therapy; psychological *adjacent to* therapy, treatment, or intervention; CBT; cognitive *adjacent to* therapy, behaviour therapy, intervention, or remediation; behaviour *adjacent to* therapy, control, modification, change, or intervention; Psychotherapy; Counseling; Social support; skills training; motivational *adjacent to* intervention, interview, or support; brief advice; brief intervention; family *adjacent to* therapy, intervention, or counselling, couple *adjacent to* therapy, intervention, or counselling; peer *adjacent to* therapy, intervention, or counselling; talking therapy; contingency management; psychoeducation; route transition; network therapy.  Key words for outcomes: Risk *adjacent to* taking, reduction, protection, minimisation, prevention, decrease, avoidance, or behaviour; harm *adjacent to* reduction, protection, minimisation, prevention, decrease, avoidance; unsafe injecting; needle sharing; intravenous injection; injecting *adjacent to* equipment, paraphernalia, or risk; sexual risk behaviour; unsafe sex; multiple sexual partners; multiple casual partners; one time sex encounter; sex holiday; casual sex; casual partner; non-regular sex partner; unprotected intercourse; unprotected sex; condomless *adjacent to* sex or intercourse; condom free *adjacent to* sex or intercourse; barebacking; bareback sex; bug chase; anal intercourse; anal sex; condom; condom use; safe sex; health *adjacent to* knowledge, attitudes, or practice, or behaviour; transmission knowledge or understanding. |
| Cochrane Collaboration | Key words for population: people who injection; people who inject drugs; PWID; Substance abuse; intravenous substance abuse; injecting drug use; intravenous drug use; IDU; injection drug use; injector; IVDU; intravenous drug abuse; injecting drug abuse; injection drug abuse.  Key words for BBV: Blood-borne pathogens, BBV , blood borne virus, blood borne bacteria, HIV infection, human immunodeficiency virus, HIV, Hepatitis C, Hepatitis C virus, hep c, HCV, Hepacivirus, Hepatitis B, Hepatitis B virus, HBV.  Key words for intervention: psychosocial *adjacent to* intervention, prevention, support, rehabilitation, treatment, or therapy; psychological *adjacent to* therapy, treatment, or intervention; CBT; cognitive *adjacent to* therapy, behaviour therapy, intervention, or remediation; behaviour *adjacent to* therapy, control, modification, change, or intervention; Psychotherapy; Counseling; Social support; skills training; motivational *adjacent to* intervention, interview, or support; brief advice; brief intervention; family *adjacent to* therapy, intervention, or counselling, couple *adjacent to* therapy, intervention, or counselling; peer *adjacent to* therapy, intervention, or counselling; talking therapy; contingency management; psychoeducation; route transition; network therapy.  Key words for outcomes: Risk *adjacent to* taking, reduction, protection, minimisation, prevention, decrease, avoidance, or behaviour; harm *adjacent to* reduction, protection, minimisation, prevention, decrease, avoidance; unsafe injecting; needle sharing; intravenous injection; injecting *adjacent to* equipment, paraphernalia, or risk; sexual risk behaviour; unsafe sex; multiple sexual partners; multiple casual partners; one time sex encounter; sex holiday; casual sex; casual partner; non-regular sex partner; unprotected intercourse; unprotected sex; condomless *adjacent to* sex or intercourse; condom free *adjacent to* sex or intercourse; barebacking; bareback sex; bug chase; anal intercourse; anal sex; condom; condom use; safe sex; health *adjacent to* knowledge, attitudes, or practice, or behaviour; transmission knowledge or understanding. |
| Clinical Trials | Key words for BBV: HIV, hepatitis,  Key words for intervention: psychosocial, behavioural |

**Table II. Description of trials included in the systematic review.**

| **Authors** | **Country** | **Outcome assessed** | **Assessment**  **(timeframe)** | **Participants** | **Intervention delivery setting/staff delivered intervention** | **Description intervention group** | | | | **Description of control group** | | **Length of follow up** | **In meta-analysis** |
| --- | --- | --- | --- | --- | --- | --- | --- | --- | --- | --- | --- | --- | --- |
|  |  |  |  |  |  | ***Intervention group/s*** | ***Number sessions*** | ***Intervention function/s*** | ***Adherence to intervention*** | ***Control intervention*** | ***Number sessions*** |  |  |
| **Abou-Saleh et al., 2008** | UK | injecting and sexual risk behaviour | Injecting Risk Questionnaire (IRQ) (past 6 months)  HIV Risk Taking Behaviour Scale (HRBD) | 95 HCV-ve PWID (26% female); mean age 32 | Outpatient drug treatment centre/ drug treatment staff | Enhanced prevention counselling (EPC)  (N=43) | 4 x 40-60 min sessions to be completed within eight weeks | Education, Enablement | There was a low adherence rate to EPC. The majority of participants who engaged only attended for one EPC session. | Simple educational counselling (SEC)  (N=52) | 1 x 10 min session | 6 months post randomisation | Yes |
| **Avants et al., 2004** | US | injecting and sexual risk behaviour | self-reported HIV risk  behaviour (penetrative sex without a condom, sharing of drug paraphernalia, and reuse of used, not bleach-disinfected,  needles). Harm reduction behaviour was assessed weekly by self-report (did/did not engage in sex without a condom or shared drug paraphernalia with and without bleach disinfecting  during the previous week). (past 3 months) | 220 PWID receiving MMT (69% female); mean age 37 | MMT/ Clinician | Standard care + Harm Reduction Group  (N=108) | 12 x 90 min weekly group sessions | Education, Enablement,  Training | 86% | Standard care + Single IMB-based HIV risk reduction session  (N=112) | 1 x 2 hr session | Post intervention | No |
| **Bertrand et al., 2015** | Canada | injecting risk behaviour | Out-come variables included dichotomous measures of injec-  tion risk behaviours deﬁned as having, in the previous  month, shared syringes, containers, ﬁlters or water and  backloading/frontloading. Primary outcome was deﬁned  as having any of those risk behaviours; each behaviour  was also examined separately, as secondary outcomes. The questions used to assess injection risk behaviours came from the NIDA Risk Behaviour Assessment (RBA) questionnaire, (past 30 days) | 219 PWID who had shared drug injection equipment or drugs by backloading or frontloading in past month (18% female); mean age 38 years | Not specified/  researcher | Motivational Intervention  (N=111) | 1 x 30–45-min MI session | Enablement,  Persuasion | 100% | Education Intervention (N=108) | 1 x 30–45 min educational intervention | 6 months post randomisation | Yes |
| **Booth et al., 2011** | US | injecting and sexual risk behaviour | Risk Behaviour Survey (RBS) assesses HIV and HCV risk behaviours in the areas of drug use and sex (past 30 days) | 632 PWID (24% female); mean age 36 | Residential detoxification centres/ interventionist | Treatment as usual (TAU) + HIV/HCV counselling and education (C&E) model  (N=212)  TAU + therapeutic alliance intervention to facilitate treatment entry  (N=209) | C&E —2 individual sessions (1^st^ 30 min session HIV pre-test counseling, and optional (although strongly encouraged) HIV testing; 2^nd^ 30 min session 2 wks later, included test results and post-test counselling).  Therapeutic Alliance (TA) —individual 45 min session | Education, Enablement,  Training  Education, Enablement | (received HIV/HCV risk assessment, HIV referral for testing, and attended HIV testing referral)  C&E- 50.1%  TAU- 36.45% | TAU: HIV/HCV risk assessment screening and referral for testing and counselling (N=211) | HIV/HCV risk assessment screening and referral for testing and counseling | 6 months post randomisation | Yes |
| **Dushay et al., 2001** | US | injecting and sexual risk behaviour | Risk Behaviour Assessment (RBA) assessed sexual behaviour and condom use, injection risk variable consists of the total number of injections in the past month, the total number of needles used by participants that were previously used by family or friends, and the total number of needles used that were previously used by strangers. The other variable summarized risky sexual behaviour (sex risk). The sex risk variable consists of the total number of sex partners in the last 30 days; the number of sex partners who were injection drug users; the total number of unprotected receptive sex acts; the number of times the respondent exchanged sex for drugs, money, or favors; and the number of times the respondent participated in sex while intoxicated with any drug, including alcohol (past 30 days) | 669 drug users [64% injecting]; (27% female), mean age 32 | Not reported/ Not reported | Ethnic cultural enhanced intervention  (n=453) | 5 sessions - 2 video sessions (1 x session 1 hr + 15 min booster 2 weeks later) + 3 x 2 hour sessions in a week | Education, Enablement, Training | 64% | AIDS knowledge and prevention video educational programme  (N=216) | 2 video sessions (1 x session 1 hr + 15 min booster 2 weeks later) | 5–10 months post randomisation (20% interviewed 12 months post randomisation) | No |
| **El-Bassell et al., 2011** | US | injecting and sexual risk behaviour | An audio computer–assisted self-interview (ACASI) was used to collect self-reported data on drug-related and sexual behavioural outcomes, including whether or not and number of times injection drugs were used and number or times syringes, cookers, cotton, or rinse water were shared with another user, number of vaginal and anal intercourse acts, number of unprotected vaginal or anal intercourse acts with their study partners and with all other partners, consistent condom use (eg, used a condom 100% of time during vaginal sex), and incidence of concurrent sexual partners (past 3 months | 282 HIV-negative drug-using heterosexual couples (564 individuals) [16% PWID]; mean age 37 | Not reported/ Trained female or male facilitator- matched to gender of the index participant | Couple-based risk reduction  (n=190)  Individual-based HIV risk reduction delivered male or female drug-involved partner (n=183) | 7 x 2 hr weekly sessions | Enablement, Training | 64% | Couple-based wellness promotion  (attention control condition)  (n=190) | 7 x 2 hr weekly sessions | 12 months post randomisation | No |
| **Gagnon et al., 2010** | Canada | injecting risk behaviour | Number of times you injected yourself and how many of those injections were given with a new syringe. The proportion of “dirty” syringes was the result of the difference between the number of injections and the number of new syringes divided by the number of injections. The behaviour was safe if the proportion of new syringes was 100% or if the number of injections was 0 (past week) | 260 PWID (31% female); mean age 35 | NEP/ Computerised intervention- audiovisual message delivered by a virtual male character | Comparison  intervention + computer-tailored messages  (N=130) | 4 x weekly sessions | Education,  Enablement, Modelling, Persuasion | 93% | needle exchanges, psychosocial support and social and health service referrals  (N=130) | 2 x 1. Each encounter lasted between 10 and 30 minutes | 3 months post intervention | No |
| **Garfein et al., 2007; Purcell et al., 2007; Mackesy-Amiti et al., 2011; 2013; 2014** | US | injecting and sexual risk behaviour | Proportion of all injections that involved: (i) injecting with a syringe used previously by another IDU, (ii) using a new sterile syringe to divide drugs with another IDU when drugs were split, (iii) sharing cookers, (iv) sharing cotton filters, (v) sharing rinse water. Response options on likert type scale from 1-never to 7-always. The drug-splitting variable had reverse coded response categories making higher values equate to greater risk. Also, assessed no. of injecting partners in the past 3 months, and the proportion with whom participants shared injection equipment with. On a likert type scale from 1= none, to 7=all. A composite injection risk variable was created by summing the 6 proportion variables and dividing by six to produce a single outcome measure with values ranging from 1 to 7 (Cronbach's alpha 0.83). Self-reported sexual behaviours: asked about number of vaginal and anal sex acts, with and without condoms, stratified by partner type (main steady, other steady, and casual/sex trade partners). Six sexual risk outcomes were computed emunerating unprotected vaginal or anal intercourse acts, with a main partner, other steady partners, and casual/sex trade partners. Summing these variables provided the total number of unprotected sex acts with all partners (past 3 months) | 654 HIV-ve and HCV-ve PWID (34% female), mean age IG 27; CG 26 (<3% enrolled participants aged 15-17 years) | Not reported/ Trained facilitators | Peer education intervention (N=431) | 6 x 2 hr sessions over 3 weeks | Education, Enablement,  Training | 56% | Video discussion group  (N=423) | 6 x 2 hr sessions over 3 weeks | 6 months post intervention | Yes |
| **Gilbert et al., 2010** | Kazak-hstan | injecting and sexual risk behaviour | Risk Behaviour Assessment (RBA). The primary behavioural outcomes in this trial were (1) the proportion of condom-protected acts of vaginal and anal intercourse, (2) the number of acts of unprotected vaginal and anal intercourse, (3) the number of incidents of unclean syringes or needles, (4) the proportion of injection acts in which unclean needles or syringes were used, and (5) the number of people with whom participants shared needles or syringes with (past 30 days) | 40 HIV-ve couples who inject drugs (50% female), mean age 32 | NEP/ facilitators | Couple Based HIV Risk Reduction Intervention  (N=40, 20 couples) | 4 x 2 hr weekly sessions (3 single-gender group sessions + an individual couple  session) | Education, Modelling, Training | Average attendance rate 99% | Attentional Control Wellness Promotion Condition  (N=40, 20 couples) | 4 x 2 hr weekly group sessions with both male and female partners | 3 months post intervention | Yes |
| **Go et al., 2013** | Vietnam | injecting and sexual risk behaviour | Self reports on the following risk behaviours: injecting drug use, sharing injecting equipment (needles, syringes, cookers, cotton and rinse water, front and back loading), properly disinfecting injecting equipment, number of sex partners, condom use during vaginal and anal sex with primary and casual partners. “used others’ needle/syringes” and “having unprotected sex” were main outcome variables to evaluate the intervention (past 3 months) | 419 index HIV-ve PWID (0% female); mean age 32 | Not reported/ Not reported | Peer network-oriented intervention  (N=210) | 6 x 2 hr weekly sessions + 3 booster sessions at 3, 6, 9 months | Education, Enablement,  Training | 60% attended all six sessions, and 30% attended all three optional booster sessions | Standard care and Government sponsored  Pamphlet on HIV  (N=209) | Standard care and Government sponsored  Pamphlet on HIV | 12 months post intervention | Yes |
| **Go et al., 2015** | Vietnam | injecting and sexual risk behaviour | Participants were asked about direct (gave or received used needles/syringes) or indirect sharing (shared injecting drugs, solutions or distilled water). Frequency of injecting, defined as the number of days injected and number of times injected per day were also assessed. For sexual risk, participants were asked if they ever had sex with a female or male sexual partner without using a condom (past 3 months) | 455 HIV-positive PWID (0% female); mean age 35 | Community intervention education sessions delivered by a trained community mobilizer; Individual HIV knowledge and skill-building group sessions conducted by a team of two facilitators | Individual level  HIV testing and counselling, plus 2 individual post-test counselling sessions, 2 small group sessions (HIV knowledge and skill-building) + optional dyad session  (N=95)  Community and individual level  Community-wide programme consisting of a 2-part video and a series of 6 HIV education sessions (N=132) | 4 individual sessions + 2 group sessions + optional dyad session  2-part video and a series of 6 HIV education sessions | Education, Enablement,  Training  Education | Among 227 participants randomized to the individual level intervention, 93%, 92%, 86% and 85% attended individual sessions 1 and 2, and group sessions 1 and 2 respectively. 83% attended all 4 sessions, and 67% attended an optional session with a support person  90% of the subjects in the community member  cohort attended the first video screening, and 82% attended the second video screening | Individual level  HIV testing and counselling  (N=89)  Community level  Standard messages on HIV through village weekly public loudspeakers and educational pamphlets already being provided by community health stations  (N=139) | 2 sessions  Time frame not specified | 24 months post randomisation | Yes |
| **Hoffman, 2013** | Russia | injecting and sexual risk behaviour | Unsafe injecting practice (combined variable including any of the following: needle or syringe sharing, shared syringe water, common dish  or common drug source) or sexual behaviour (condom use past 30 days; number of partners past 3 months) | 432 PWID (33% female); mean age 29 | Research centre/ session facilitators | Peer educator training intervention  (N=99)  Network members (N=127) | 8 training sessions (7 group + 1 individual training) + booster meeting once a month for 4 months | Education, Modelling, Persuasion, Training | Median number of training sessions attended was  7 (IQR 3, 8) | Group sessions devoted to areas of interest  (N=92)  Network members (N=114) | 8 sessions (all group) of equivalent length. No booster | 24 months post randomisation | No |
| **Latka et al. 2008; Kapadia et al, 2007** | US | injecting risk behaviour | Outcomes measured by self-report using audio-computer assisted self-interviews (ACASI), incl: 1. syringe lending (measured by frequency of passing a used syringe to another - on a 7 point scale from always to never) 2. unsafe drug preparation (measured by frequency of dividing up drugs with a syringe one had previously used - on a 7 point scale from always to never). 3. sharing drug preparation equipment - 3 questions about frequency of using cookers, cottons, or rinse water with or before someone else, combined into a single dichotomous variable any/no sharing 4. whether injected - self-report to question about drugs injected, coded as dichotomous variable injected/did not inject. A single primary outcome variable comprising the previous 4 variables. Secondary outcomes: 1. whether participants had refrained from lending syringes because they had HCV (single dichotomously coded item). 2. participation in drug treatment since prior study visit (includes biomedical treatment and support groups) was a dichotomized variable. 3. frequency of injecting oneself using a used syringe - single item. (past 3 months) | 418 HCV+ve PWID (24% female), mean age 27 | 3 research sites/ trained facilitators | Peer-mentoring behavioural intervention (N=222) | 6 x 2 hr sessions held twice weekly | Education,  Training | 46-70% attended all 6 sessions (multi-site study) | Time-equivalent attention-control (video-discussion) group (N=196) | 6 x 2 hr sessions held twice weekly | 6 months post intervention | Yes |
| **Latkin et al., 2003** | US | injecting and sexual risk behaviour | To assess needle sharing, we asked participants, “In the past six months, when you injected (shot up) drugs, how often did you use a needle or tools  [injection equipment] immediately after another person used it, without cleaning it first with bleach?” The eight response options ranged from *never* to *more than once a day*. Stopping drug injection was defined as reporting any illicit drug injection. Frequency of condom use during vaginal, oral, and anal sex with their main  and casual partners. Frequency of condom use was assessed with a 5-point scale, using the response categories *never, less than half the time, half the time, more than half the time*, and *always; every single time* (past 6 months) | 250 [47% PWID] (39% female); mean age 39 | Clinic/ indigenous para-professional facilitators | Multisession, small-group experimental condition, which encouraged peer outreach  (N=81) | 10 x 90 min small group training sessions | Enablement, Education,  Training | Mean 6.5/10 sessions attended | Equal-attention control condition (N=36) | 10 x 90 min sessions | 6 months post intervention | Yes |
| **Latkin et al., 2009; Latkin et al., 2013** | Thailand and US | injecting and sexual risk behaviour | Frequency of injection drug use, sharing injection equipment (needles, cookers, cotton, and rinse water, front and back loading (i.e. injecting drugs from one syringe to another)), properly disinfecting injection equipment, condom use during vaginal and anal sex with primary and casual partners, HIV prevention conversations, and the number of sex partners (past 30 days) | 414 networks with 1123 HIV-ve participants [91% PWID (94% in US & 88% in Thailand)] (3% females in Thailand, mean age 32; 20% females in US, mean age 41) | Not reported/ facilitator | HIV counseling and testing + small group peer-educator sessions  (N=204 networks) | 2 individual sessions +  6 x 2 hour small-group sessions over 4 weeks + 2 booster sessions at 6 and 12 months | Education,  Enablement, Persuasion, Training | 72% in US and 96% in Thailand attended at least 4 sessions | HIV counseling and testing (N=210 networks) | 2 individual sessions | Up to 30 months (24 in Thailand) post randomisation | Yes |
| **Margolin et al., 2003** | US | injecting and sexual risk behaviour | Two categorical measures of risk behaviours- needle sharing and unprotected penetrative sex- asked patients if they had engaged in either of these two behaviours (0=no, 1=yes) at Time 1, since learning their HIV-seropositive status; at Time 2, during the 6-month treatment, and at Time 3, during the 3-month posttreatment follow-up.  To assess a broader range of drug- and sex- related HIV risk behaviours over equivalent time periods the Risk Assessment Battery (past 30 days) | 90 PWID HIV+ve entering MMT (30% female); mean age 41 | MMT/ counsellor | Enhanced-MMT (E-MMP)  6 months of standard treatment  (daily methadone and weekly individual substance abuse counseling and  case management) enhanced by the inclusion of a six-session HIV risk  reduction intervention  (N=45) | 6 months of standard treatment: Daily methadone + weekly individual substance abuse counseling + case management; 6-session HIV risk reduction intervention + twice weekly x 2 hr manual-guided group therapy sessions | Enablement, Persuasion,  Training | 64% completed 6-month programme | Enhanced-MMT (E-MMP)  6 months of standard treatment  (daily methadone and weekly individual substance abuse counseling and  case management) enhanced by the inclusion of a six-session HIV risk  reduction intervention (N=45) | 6 months of standard treatment: Daily methadone + weekly individual substance abuse counseling + case management; 6-session HIV risk reduction intervention | 9 months post randomisation | Yes |
| **McMahon et al., 2013; 2015** | US | injecting and sexual risk behaviour | Questions on dyadic (within-couple) risk, such as HIV testing behaviour, engagement in vaginal and anal intercourse, frequency of condom use, injection and non-injection drug use, and conception desires (past 3 months) | 330 HIV-ve drug users (100% female) and primary male partners [48% of women PWID]; mean age 39 [Couples IG & CG] and 38 [Women only IG] | Field office/ interventionist | (1) Couple-Based HIV Counseling and Testing Intervention (N=110) [43% PWID]  (2) Women only  relationship-focused HIV counseling and testing (N=104) [51% PWID] | 2 sessions  2 sessions | Education, Enablement | Not reported | NIDA  standard HIV- counseling and testing (N=116) [51% PWID] | 2 sessions | 9 months post intervention | No |
| **Otiashvili et al., 2012** | Georgia | injecting risk behaviour | HIV drug-risk was measured using a modified HIV Risk Assessment Battery (RAB), the Baltimore RAB (BRAB) The BRAB includes 11 items that measure HIV drug risk behaviour. Drug-risk scores can range from 0 to 29, with higher scores indicating greater HIV drug-risk behaviour (past 30 days) | 40 drug users (0% female) [98% PWID] and their drug-free female partners; mean age 36 | Research Unit/ Counsellor | Motivational Interviewing for male participant and couple, contingency mangement + naltrexone  (n=20)  Participants have option to enter 14-day detox shortly after study entry  Female partners also asked to attend couples counselling sessions during weeks 7-18 of treatment | 22 weekly sessions | Enablement, Incentivisation | Mean number of treatment sessions attended 12.1 | Education sessions. Referrals to detox programme and aftercare that could or could not have included naltrexone  (n=20) | 22 weekly sessions | 6 months post intervention | Yes |
| **Purcell et al., 2004; 2007** | US | injecting and sexual risk behaviour | A-CASI (audio computer-assisted self interview. The outcome variable for sexual risk was dichotomized to indicate whether or not participants reported any unprotected vaginal/anal sex with their HIV-negative or unknown status partners. The key injection risk behaviour outcome was whether, in the past 3 months, the participant had lent a syringe that he or she had previously used to any HIV-negative or unknown serostatus partners or whether the participant had shared cottons, cookers, or rinse water with any HIV-negative or unknown serostatus partners. (past 3 months) | 966 HIV +ve PWID in past year (36% female), mean age 42 | Not reported/ Male and female facilitator (peers) | Peer mentoring intervention  (N=486) | 10 sessions (twice a week for 5 weeks - 7 group sessions; 2 individual sessions; and 1 ‘peer volunteer activity’) | Education, Enablement, Training | Average session attendance 82% (range: 67% to 100%) | Video Discussion intervention (N=480) | 8 group sessions over 5 weeks | 12 months post intervention | Yes |
| **Robles et al 2004** | Puerto Rico | injecting risk behaviour | Frequency of use of shooting galleries, and sharing of drug-use equipment such as needles, cottons, and water. Drug use patterns and HIV risk behaviours were assessed using revised and culturally adapted versions of the Risk Behaviour Assessment (RBA) and Risk Behaviour Followup Assessment (RBFA) instruments (past 30 days and past 6 months) | 557 PWID (4% female); mean age not reported | Assessment facility or drug treatment/ registered nurse | Standard intervention (2 one-on-one sessions on HIV/AIDS risk)+ 6 counseling sessions + case management  (N=285) | 6 weekly sessions | Education, Enablement, Training | 67% | Standard intervention: 2 one-on-one sessions on HIV/AIDS risk (N=272) | 2 sessions | 6 months post randomisation | Yes |
| **Rotheram-Borus et al., 2010; Herschberger et al., 2003** | US | injecting and sexual risk behaviour | Counts of the number of vaginal and anal sexual acts, and the number of these sexual acts that were unprotected. Safer sex was defined as 100% condom use or abstinence. For injection drug use, participants also reported the number of times they injected with “dirty” (needles/syringes) that had not been cleaned with bleach (past 30 days) | 1116 drug users (65% PWID) (33% female), mean age 38 | Field office/Counsellors and street outreach workers | HIV counseling and testing  + Psycho-educational HIV prevention programme (2 small-group skills focused workshops = 1 individual counselling session + 2 street hang outs with outreach workers + attend at least 2 monthly social events)  (N=558; 359 PWID) | 2 sessions HIV counseling and testing  + 5 sessions over 4 months (2  small-group, skill-focused workshops & 1 individual  counseling session + engagement at least 2 sessions of structured contact with  outreach workers/attendance at monthly social events) | Enablement,  Training | Not reported | HIV counseling and testing  (N=559; 364 PWID) | 2 sessions | 9 months post randomisation | Yes |
| **Samet et al., 2015** | Russia | injecting and sexual risk behaviour | unprotected sex acts (past 3 months)  Any needle sharing was defined as distributive or receptive sharing in the past 30 days captured through the Risk Behaviour Survey (RBS) (past 30 days) | 700 HIV-infected with past 6 months risky sex and heavy alcohol consumption (60% PWID) (41% female), mean age 30 | Hospital setting/ peer interventionists | Healthy relationships intervention  2 individual and 3 small group sessions (n=350; 212 PWID) | 2 individual sessions and 3 small group sessions stressing disclosure of HIV serostatus and condom use | Education, Enablement, Modelling,  Training | IG 52% full attendance, 29% partial attendance | Attention control  2 individual and 3 group sessions  (n=350; 211 PWID) | 2 individual sessions and 3 group sessions focused on stress reduction, social support and good nutrition for HIV-infected individuals | 12 months post randomisation | Yes |
| **Schroeder et al, 2006; Esptein et al 2003** | US | injecting and sexual risk behaviour | Outcomes were frequency of (i) risky drug related behaviour, and (ii) risky sexual behaviour, in the past week. Drug related behaviours included how often the participant injected any kind of drug, and how frequently s/he injected with needles that had previously been used (either cleaned with bleach, or "dirty" needles). Sexual behaviours included how frequently the participant had unprotected sex (with and IV drug user, with someone s/he didn't know well, or that involved anal sex), and how frequently s/he traded sex for money, drugs, or gifts. All responses were dichotomised to none vs some (ie. any occurrence of the behaviour). (past week) | 81 cocaine and heroin dependent drug users [96% PWID] (female 52%); mean age 38 | Outpatient research clinic/ counsellor | Standard dose of methadone (70-80mg/day) + + weekly individual counselling (5 weeks) followed by 12 weeks of intervention condition, followed by a 12-week standard treatment  (1) weekly CBT + Contingent vouchers (CM)  (N=16)  (2) weekly CBT + noncontingent vouchers  (N=19)  (3) CM plus weekly group therapy  (N=22) | 29 weeks (methadone + 5 weekly individual counselling , 12 weeks of intervention, followed by 12 weeks standard treatment | Education, Enablement, Incentivisation  Modelling, Training | 77% completed the 12-week behavioural intervention phase (63% CBT only – 88% CBT+CM) | Standard dose of methadone (70-80mg/day) + + weekly individual counselling (5 weeks) followed by 12 weeks of control condition, followed by a 12-week standard treatment  Group therapy + non-contingent vouchers  (N=24) | 29 weeks (methadone + 5 weekly individual counselling , 12 weeks of intervention, followed by 12 weeks standard treatment | Post intervention | Yes |
| **Stein et al., 2002** | US | injecting risk behaviour | HIV drug risk behaviour was measured as the number of days subjects reported sharing works. An injection-related HIV risk behaviour day was defined as one on which the study participant responded positively to the question, ‘‘Have you used needles, cotton, or a cooker after someone else had used it, even if you cleaned it with bleach before you used it?’’ Primary outcome was the number of on which IRRB occurred at 6-month follow up. We also constructed a dichotomous indicator that contrasts the presence or absence of IRRB days at 6-months. We also constructed dichotomous indicators giving relative reductions in IRRB days of 25% or more, 50% or more, and 75% or more. Additionally, we constructed a dichotomous indicator giving absolute change in IRRB days of one or more (past 30 days) | 109 PWID who were also hazardous drinkers (38% female); mean age 36 | Needle exchange attenders attended research site out with NEP/ social worker | Standard list of referrals for substance abuse and medical treatment + Brief motivational intervention  (N=60) | 2 sessions one month apart (1^st^ session 60 mins, 2^nd^ session 30–45 mins) | Enablement | 95% | Standard list of referrals for substance abuse and medical treatment provided  (N=49) | n/a | 6 months post randomisation | Yes |
| **Stein et al., 2005** | US | injecting risk behaviour | Drug-risk component of the HIV Risk Assessment Battery. This measure was comprised of 8 items; the scoring protocol generates scale scores ranging from 0 to 1.0 with high scores indicating relatively higher levels of HIV drug-risk behaviour (past 30 days) | 109 PWID (36% female); mean age 37 IG, 36 CG | Outpatient academic research office/ Clinical Psychologist | CBT + pharmacotherapy for treatment of depression. No sessions were specifically devoted to cessation of drug use  (N=53) | 8 x 1 hr CBT visits (over 3 months) and 3 pharmacotherapy visits (monthly over 3 months) | Education,  Enablement, Training | attended mean 4.04 (+3.15) of 8 CBT appointments; participants took medications on 39.4% of the days they were prescribed; 43% ‘‘highly adherent’’ to treatment | Assessment only  (N=56) | Assessment visit | 9 months post randomisation | Yes |
| **Stein et al., 2009** | US | injecting risk behaviour | Drug equipment sharing behaviours was assessed at each interview with the question, “What is the number of times you shared needles or works?” (past 6 months) | 277 HCV-ve out-of-treatment heroin and/or cocaine users [28% PWID] (37% female); mean age 37 | Not stated/ interventionist | Motivational Interview  (N=140) | 4 sessions x 30-45 mins at baseline, 1, 3, and 6-months post-baseline | Education, Enablement, Persuasion | Not reported | Information handout about local treatment resources  (N=137) | n/a | 24 months post randomisation | No |
| **Sterk et al., 2003** | US | injecting and sexual risk behaviour | Number of injections with used needles/syringes; number of times works or water was shared; number of injections (past 30 days) | 68 HIV-ve PWID (100% female) mean age 41 | Health Intervention Project House/ Female health interventionists | Enhanced motivation intervention  [EMI] (N=20)  Enhanced negotiation intervention  [ENI] (N=21) | Enhanced motivation intervention: 4 sessions  Enhanced negotiation intervention: 4 sessions | Education, Enablement, Training | 95% in motivation condition, and 100%  in negotiation condition | NIDA standard intervention (excluding impact of race and gender on HIV risk behaviours)  (N=27) | 2 sessions | 6-months (not clear if post intervention or post randomisation) | Yes |
| **Strathdee et al., 2013; Vera et al., 2012** | Mexico | injecting and sexual risk behaviour | Secondary outcomes included frequency of receptive needle sharing, and sharing of injection paraphernalia (cookers, cottons, water and dividing drugs with a used syringe). Also, Injection risk index score (comprised of the following: receptive needle sharing, sharing a cooker, cotton filter, or rinse water to prepare drugs for injection after someone else had used it, and using a used syringe to divide drugs. The score was constructed by calculating the average between the responses to these five injection risk indicators (1=never, 2=sometimes, 3=about half the time, 4=often, and 5=always), with higher scores representing higher risk (past 30 days and past year) | 584 Sex Workers who inject drugs (100% female) median age  -Tijuana 34, Ciudad Juarez 33 | Not reported/ female bi-cultural counselors | Interactive Injection Risk Intervention and Didactic Sexual Risk Intervention  (N=146)  Interactive Sexual Risk Intervention Condition and Didactic Injection Risk Intervention Condition  (N=148)  Interactive Injection Risk Intervention and Interactive Sexual Risk Intervention)  (N=146) | 1 x 60 min session | Education, Enablement, Modelling,  Persuasion, Training | not reported | Didactic Injection Risk Intervention and Didactic Sexual Risk Intervention) (N=144) | 1 x 60 min session | 12 months post randomisation | No |
| **Tobin et al., 2011** | US | injecting and sexual risk behaviour | Injection and drug-splitting risk. All participants were asked regarding frequency of use of these paraphernalia. Injection risk behaviour was operationalized as any use (versus never) of any unclean paraphernalia. Drug-splitting risk was based on having shared a cooker when preparing drugs versus not. Sex risk. All participants (Index and RNM) reported the total number and type (main versus non-main) of sex partners. A dichotomous variable was created based on the frequency of condom use for anal or vaginal sex with any type of sex partner defined as 100% condom use for both vaginal and anal or less than 100% for either. Engaging in sex risk behaviour was operationalized as: (i) two or more sex partners (ii) having sex in exchange for money, drugs, food or shelter; or (iii) less than 100% condom use for either vaginal or anal sex with any type of sex partner. A dichotomous variable was constructed to indicate engaging in at least one of the sex risk behaviours versus not (past 6 months)  The number of different people with whom the participants had shared cookers or needles (past 30 days) | 227 PWID (45% female), mean age 43 | Research clinic/ Not reported | Peer educator intervention  (N=114) | 7 session (5 group-based, one individual and one session with Index participant and their enrolled Risk Network Members) | Education, Enablement, Training | 87% attended at least 4/7 sessions; 36% attended all sessions, and 64% completed the dyad session. | Group information sessions (N=113) | 5 group information sessions | 18 months post intervention | Yes |
| **Tucker et al., 2004** | Australia | injecting and sexual risk behaviour | BBV-TRAQ is a standardized instrument consisting of 34 items, with three subscales measuring the prevalence of injecting, sexual and skin penetration risk practices (past 30 days) | 145 PWID  (26% female); mean age 31 | Specialist out-patient clinical and research organization/ clinical researcher | Tailored brief behavioural intervention  (N=73) | 1 x 30 minute session | Education, Enablement | 100% | Written educational materials regarding HCV  (N=72) | n/a | 1 month post randomisation | Yes |
| **Wechsberg et al., 2012** | Russia | injecting and sexual risk behaviour | Russian adapted Revised Risk Behaviour Assessment (RRBA) for women. Injection-related HIV-risk behaviours (i.e., receptive syringe sharing, indirect sharing [sharing of cookers and cottons], and syringe-mediated drug sharing [sharing liquefied drugs that have been prepared with water added from a used syringe]) and counts of oral, vaginal and anal sex behaviours, as well as the number of episodes where condoms are used were assessed (past 30 days) | 100 PWID (100% female); mean age 26; 57% HIV+ve | Inpatient detoxification/ Female Psychologist | Woman-Focused Intervention  (N= 51) | 2 x 1 hr weekly sessions | Education, Enablement, Training | Not reported | Nutrition Intervention (N=49) | 2 x 1 hr weekly sessions | 3 months post randomisation | Yes |
| **Zule et al., 2009** | US | injecting and sexual risk behaviour | Use of a new syringe at last injection and condom use at last sexual encounter were selected to assess secondary outcomes for injection and sexual risk reduction (last injection and last sexual encounter) | 851 PWID (27% female); mean age  41 (of those who completed one or more follow-ups) | Not reported/ Trained lay persons from the community | Motivational Intervention for HCV risk reduction  (n=423) | 6 sessions | Education, Enablement,  Modelling, Persuasion | Range from 100% (session 1) -36% (session 6) | Video Educational  Intervention for HCV risk reduction  (n=428) | 6 sessions | 12 months post randomisation | Yes |
